# Assessment of Churn in Coverage Among California’s Health Insurance Marketplace Enrollees

**DOI:** 10.1001/jamahealthforum.2022.4484

**Published:** 2022-12-02

**Authors:** Emory Wolf, Mary Slosar, Isaac Menashe

**Affiliations:** 1 Covered California, Sacramento, Sacramento; 2 Slosar Research, Middlebury, Vermont

## Abstract

**Importance:**

Individual health insurance marketplaces established through the Affordable Care Act of 2010 (ACA) fill a critical gap for those who lack other coverage options. The high degree of coverage turnover, or churn, has raised concerns about affordability and strategic behavior on the part of individuals to sign up only when they need care.

**Objective:**

To assess the role of California’s ACA marketplaces in the broader health care landscape by understanding enrollment tenure and churn.

**Design, Setting, and Participants:**

This cross-sectional study analyzed survey data from a representative, probability-based sample of enrollees in California’s ACA marketplace, Covered California, collected immediately after the open enrollment periods in 2018, 2019, and 2021. Administrative data from Covered California from January 1, 2014, to December 31, 2021, were used to analyze marketplace tenure. Survey data included 9571 heads of households aged 18 to 64 years who were newly enrolled or had recently terminated their plan, directly drawn from Covered California’s administrative records. Administrative data included individuals up to age 65 years who enrolled in the marketplace during 2014 to 2021.

**Exposures:**

New enrollment in or termination from health care coverage through California’s ACA marketplace.

**Main Outcomes and Measures:**

Enrollment tenure in California’s ACA marketplace, sources of coverage prior to enrolling and after terminating coverage, and demographic or plan characteristics associated with the decision to go uninsured.

**Results:**

Median (IQR) length of coverage among 5.4 million enrollees (mean [SD] age, 38 [16] years; 17% Asian American/Native Hawaiian or other Pacific Islander, 2.5% Black or African American, 23% Latino [response options were Hispanic, Spanish, or Latino origin], 29% White, 7.5% categorized as other [including American Indian/Alaskan Native, multiple races, and other], and 21% of unknown race or ethnicity) was 14 (6-35) months, and 41% to 46% of enrollees disenrolled within 1 year, with substantial variation by subgroups. Despite this churn, only 14% (95% CI, 12%-15%) of 6474 terminating members surveyed across 3 years (2018, 2019, and 2021) reported being uninsured after leaving the marketplace, with the rest moving to job-based coverage or Medicaid. Most of those surveyed (mean [SE] percentage, 56% [0.016] individuals) reported having had employer-sponsored insurance or Medicaid prior to enrolling in the marketplace. Among subsidized renewal candidates, Latino candidates were 1.5 percentage points (95% CI, 0.8-2.3 percentage points) more likely to go uninsured compared with White candidates, and those with no expected physician visits in the coming year were 4.8 percentage points (95% CI, 2.4-7.2 percentage points) more likely to go uninsured vs those who expected physician visits.

**Conclusions and Relevance:**

The results of this cross-sectional study of coverage churn found that ACA marketplaces served 2 distinct types of individuals, long-term enrollees but, more often, individuals with short-term–coverage needs due to a change in eligibility for other insurance. These results suggest that marketplaces are smoothing coverage disruptions and that policies to reduce gaps in coverage should be designed with this in mind.

## Key Points

**Question:**

What factors are associated with enrollment turnover, or churn, in the individual insurance marketplaces?

**Findings:**

In this cross-sectional study of 5.4 million enrollees in California’s health insurance marketplace, from 2014 through 2021, many had short enrollment tenures, with a median tenure of 14 months. Survey data from 6474 members who terminated coverage in 2018, 2019, or 2021 indicated that most churn was associated with changes in eligibility; most disenrolled individuals took up other types of coverage (46% through an employer and 24% through Medicaid), with only 14% going uninsured.

**Meaning:**

This study found that health insurance marketplace churn was largely the result of changes in eligibility to other sources of coverage rather than enrollees taking up coverage only when they needed care, suggesting that marketplaces should adopt policies to smooth the high volume of coverage transitions among its enrollees.

## Teaser

This cross-sectional study used administrative data from California’s Affordable Care Act marketplace, Covered California, and survey data from a representative sample of enrollees to identify factors associated with enrollment turnover, or churn, in the individual insurance marketplaces.

## Introduction

Twelve years after of the passage of the Patient Protection and Affordable Care Act of 2010 (ACA), the national nonelderly uninsured rate has fallen, from 17% in 2013^1^ to 10% in 2021,^2^ and many policy makers seek to build on this success for the next phase of health care reform.^3^ Although Medicaid expansion has played a large role in this reduction, the individual health insurance marketplaces have provided a pathway for access to high-quality coverage and financial help to pay for it.

Despite success enrolling many long-term uninsured initially, the marketplaces experience substantial turnover, or churn, with many individuals terminating their coverage each year.^4,5^ Data from the Centers for Medicare & Medicaid Services indicate that churn in the federally facilitated marketplace (HealthCare.gov) is high, with 35% of enrollees in 2015 terminating their coverage by December of that year.^6^ Much of this is attributable to marketplace enrollees having short enrollment tenures, with some analyses showing that 35% to 40% of enrollees terminate their coverage within 1 year.^6,7^

At first glance, this rate of terminations from marketplace coverage is concerning. Unlike those with employer-sponsored insurance (ESI) or Medicaid, consumers enrolled in the marketplace elect to pay for their own coverage or go uninsured. If enrollees are ending their coverage due to rising costs or shortly after obtaining any needed care, the sustainability of the risk pool could be in jeopardy.^8,9^

However, there is little research documenting the sources of coverage for those who enter and leave the marketplaces. Preliminary evidence,^8^ including an exit survey of consumers using the federal HealthCare.gov platform that found that 51% of enrollees who terminated their marketplace coverage did not obtain coverage elsewhere, reinforces the concern that the marketplaces may be unaffordable for those without other coverage options.

Alternatively, turnover may be a sign that the marketplaces are working as expected, serving consumers during coverage disruptions; these enrollees may sign up upon losing coverage but then terminate coverage when they become eligible for insurance elsewhere (eg, through a new job, Medicaid, or Medicare). For this population experiencing life changes, the marketplaces provide continuity in a fragmented system of coverage.

This study aimed to assess the roles of the marketplaces by examining churn dynamics in California’s state-based marketplace, Covered California.^10^ To our knowledge, this is the first study to link marketplace administrative data with survey responses on sources of coverage to examine coverage dynamics for consumers experiencing marketplace churn and to review the possible implications for future policies to expand coverage.

## Methods

This cross-sectional study was approved by the California Health and Human Services Agency Institutional Review Board, and a waiver of the need for informed consent was granted because the survey was not considered human participants research. The administrative data contain protected personal information and are not deidentified, but no patient-identifying information is reported in this study. The study followed the Strengthening the Reporting of Observational Studies in Epidemiology (STROBE) reporting guidelines on cross-sectional studies.

The main outcomes were (1) variation in marketplace enrollment and tenure, assessed using administrative data; (2) enrollees’ sources of coverage before enrollment and after disenrollment from the marketplace, assessed using representative survey data from Covered California enrollees; and (3) consumers’ reasons for deciding to go uninsured rather than renew coverage, assessed using the survey data.

### Administrative Data

We drew on Covered California administrative data for information on enrollment and tenure, analyzing enrollee-level data from plan years 2014 through 2021 (January 1 through December 31 for each year) that included information on eligibility, demographic characteristics, and plan attributes as of the initial enrollment. We excluded consumers older than 64 (0.57% of enrollees) years and anyone missing any age, plan, or demographic attributes (fewer than 0.01% of enrollees).

### Survey Data

We relied on survey data from a representative, probability-based sample of current and former Covered California enrollees from the 2018, 2019, and 2021 open enrollment periods. The survey data included 9571 heads of households aged 18 to 64 years who were newly enrolled or had recently terminated their plan. Survey data following the 2020 open enrollment period were excluded from this analysis due to unique circumstances related to the onset of the COVID-19 pandemic that raised concerns about data validity. Conducted immediately after each open enrollment period since 2018 by the nonpartisan research organization NORC at the University of Chicago on behalf of Covered California, the California Health Coverage Survey uses mailed invitations for a web-based survey and is administered in English and Spanish to heads of households aged 18 to 64 years selected from a sample of Covered California’s administrative data. All survey data are weighted to population control totals from the administrative data, and survey responses are linked to administrative records for each respondent. Additional details about the survey fielding, data preparation, and survey items used in our analysis are provided in eAppendix 2 and eTables 3 through 6 in Supplement 1.

### Tenure of Coverage

We assessed patterns of tenure by examining lengths of continuous coverage, stratified by the year of the enrollee’s first month of coverage. To address time-based censoring (because mean tenure for members who are still enrolled remains unknown), we assessed the mean (SE) monthly termination rate and the median (IQR) months of coverage. We documented subgroup differences that were associated with shorter and longer enrollment tenure using a range of eligibility, income, and health plan attributes and formally estimated hazard ratios (HRs) between subgroups by fitting a Cox proportional hazards model, defining the hazard as termination, defining time as months of coverage, and considering as censored any enrollee still enrolled in December 2021. Additional details of the modeling approach and covariates used for the survival analysis, along with alternate specifications, are provided in eAppendix 1 and eTables 1 and 2 in Supplement 1.

### Coverage Sources Before Enrolling and After Leaving the Marketplace

Using the survey data collected by NORC, we conducted a descriptive analysis of postmarketplace coverage among terminating members and prior main sources of coverage (if any) among new enrollees. For terminating members, the results reflect respondents’ reported health care coverage status immediately following the open enrollment after their coverage ended. For example, the 2018 survey results reflect the coverage status at the time of the survey (roughly April 2018) among 2017 members who ended their coverage before January 1, 2018. New members were asked to report their main source of coverage in the year prior to signing up for marketplace coverage. Only individuals who reported not having coverage for the entire year prior to enrolling were recorded as uninsured.

### Renewal Candidates Who Went Uninsured

To better understand the decision to drop coverage and go uninsured, we estimated the binary outcome of going uninsured vs reenrolling during the 2019 open enrollment period using binary logistic regression.

We focused on the subset of consumers (2019 renewal candidates) who faced the decision of whether to renew their coverage or go uninsured during the 2019 open enrollment period by excluding those who reported another source of coverage for 2019. We limited the analysis to those who had incomes between 138% and 400% of the federal poverty level (FPL) and thus qualified for subsidies that lowered their monthly premium in 2018; 88.5% (95% CI, 86.2%-90.5%) of renewal candidates were receiving subsidy in 2018 (n = 2130). Unsubsidized enrollees may face a different set of priorities in their renewal decisions, given that they experience annual premium increases differently from subsidized consumers.

To explore the hypothesis that affordability and plan dissatisfaction were associated with the decision to drop coverage, we included variables that measure percentage change in net premium from 2018 to 2019, expected number of physician visits in the next year, and enrollees’ perceived value of their 2018 plan, with the expectation that those who faced high cost increases, viewed their plans as having low value, and had low expected health care use were more likely to terminate and go uninsured. We explored other possible motivations, such as awareness of the individual mandate penalty repeal, those with a history of coverage gaps in 2018, and those in low actuarial value metal tiers (plans such as bronze, that have higher deductibles and greater out-of-pocket costs^11^), all potentially being more likely to lead to dropped coverage. These factors address a hypothesis that certain consumer segments enroll in low-cost coverage, possibly in response to the penalty or only when they believe they need care. We included other demographic control variables, including race and ethnicity (self-reported by individuals though the Covered California application), FPL, age, education, and region. Additional details and iterations of the model are provided in eAppendix 3 and eTables 10 through 13 in Supplement 1.

### Statistical Analysis

All data were analyzed in 2021 and 2022 using Stata software, version 17 (StataCorp LLC), with svy suite commands used for analysis of survey data. Both Cox proportional hazards and survey estimates are reported with 95% CIs, and *P* < .05 was considered statistically significant. All tests were 2-sided.

## Results

### Tenure Among Marketplace Enrollees

**Figure 1.**
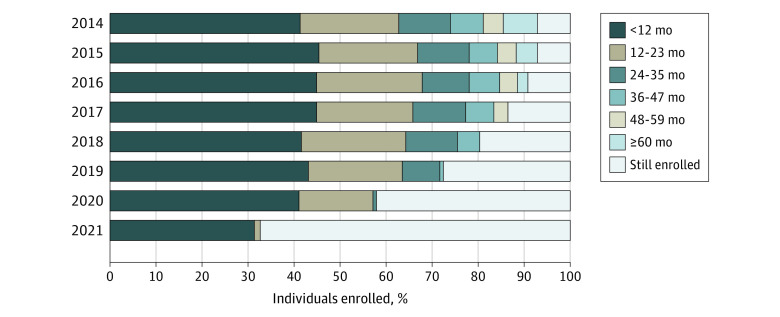
Membership Tenure Among New Marketplace Cohorts, by Enrollment Year Data included all members with continuous enrollment that began at some point during the year in question (2014: n = 1 120 320; 2015: n = 678 552; 2016: n = 593 442; 2017: n = 520 356; 2018: n = 536 821; 2019: n = 437 444; 2020: n = 717 964; 2021 = 671 678). Members with effectuated enrollment in January 2022 as part of a single, continuous coverage period that began in the year in question were considered still enrolled.

The administrative data showed that among 5.4 million enrollees (mean [SD] age, 38 [16] years; 17% Asian American/Native Hawaiian or other Pacific Islander, 2.5% Black or African American, 23% Latino [response options were Hispanic, Spanish, or Latino origin], 29% White, 7.5% categorized as other [including American Indian/Alaskan Native, multiple races, and other], and 21% of unknown race or ethnicity), representing 6.5 million discrete segments of active coverage in California’s health insurance marketplace from 2014 to 2021, the median (IQR) enrollment duration was 14 (6-35) months (Table 1). When looking at segments of continuous enrollment, 41% (2014, 2018, 2020) to 45% (2015, 2016, 2017) of individuals left the marketplace less than 12 months after enrolling (Figure 1). However, the remaining enrollees maintained their marketplace coverage for longer periods, with 53% (2015 and 2016) to 58% (2014 and 2018) enrolling for more than 12 months, and 30% (2016) to 37% (2014) enrolling for 24 or more months.

**Table 1.** Covered California Disenrollment Rates, Median Tenure, and Proportional Hazards of Coverage Terminations

	Descriptive data on tenure			Cox proportional hazards model of coverage terminations^a^
	Distribution of enrollment weighted by months of coverage, %	Monthly termination rate, %	Median tenure, mo (IQR)	HR (95% CI)
All enrollees	100	3.9	14 (6-35)	
Age, y				
0-17	7	4.4	12 (5-32)	1.197 (1.193-1.202)
18-29	19	5.1	12 (6-26)	1.287 (1.284-1.290)
30-44	24	4.3	12 (6-32)	1.230 (1.227-1.233)
45-64	49	3.2	19 (8-45)	1 [Reference]
Race and ethnicity				
Asian American/Native Hawaiian or other Pacific Islander	19	3.3	17 (8-43)	0.967 (0.964-0.970)
Black or African American	2	5.9	10 (4-22)	1.379 (1.372-1.387)
Latino	22	4.4	12 (6-32)	1.104 (1.101-1.107)
White	30	3.8	14 (7-36)	1 [Reference]
Other^b^	7	3.9	14 (6-36)	1.008 (1.004-1.012)
No response	20	4.0	13 (6-34)	1.069 (1.066-1.072)
Language spoken				
Asian or Pacific Islander languages	8	2.8	22 (10-53)	0.868 (0.864-0.872)
English	78	4.0	13 (6-34)	1 [Reference]
Spanish	11	3.7	15 (8-36)	0.939 (0.936-0.943)
Other	3	4.7	12 (6-27)	1.183 (1.177-1.189)
Income group, % of FPL				
≤150	17	4.3	12 (6-32)	1.197 (1.194-1.201)
>150-200	32	3.7	15 (7-36)	1.017 (1.015-1.020)
>200-400	42	3.8	15 (7-36)	1 [Reference]
>400 or unsubsidized	11	4.7	11 (5-29)	1.038 (1.034-1.042)
Health plan metal tier^c^				
Minimum coverage	1	8.5	8 (3-15)	1.437 (1.427-1.447)
Bronze	27	4.2	13 (6-34)	1.119 (1.117-1.122)
Silver	60	3.7	15 (7-36)	1 [Reference]
Gold or platinum	12	4.0	13 (6-35)	0.976 (0.973-0.979)
Additional factors				
Broker assistance	48	3.5	17 (7-41)	0.865 (0.863-0.867)
Special enrollment period	24	4.9	12 (5-30)	1.192 (1.190-1.195)
Monthly premium $1.00 or less	14	2.5	24 (10-69)	0.589 (0.587-0.591)
Household referred from Medicaid eligibility system	40	4.9	18 (8-42)	0.758 (0.756-0.759)
Self-employment income	28	2.3	29 (11-65)	0.557 (0.556-0.558)

Abbreviations: FPL, federal poverty level.

^a^ For the Cox proportional hazards model, additional demographic control variables included but not displayed include region, gender (transgender male and female individuals were rolled into the male and female categories, respectively), and household size. See eTable 2 in Supplement 1 for details, as well as alternate model specifications.

^b^ Other included American Indian/Alaskan Native, multiple races, and other.

^c^ Metal tier refers to level of benefits provided by a plan, according to actuarial value: a bronze plan covers, on average, 60% of annual costs; a silver plan covers 70%, 73%, 87%, or 94% of costs (participation is restricted according to income level); a gold plan covers 80% of costs; and a platinum plan covers 90% of costs.^11^

Both the descriptive measures of tenure and the survival analysis revealed substantial variation by demographic subgroups and marketplace attributes (Table 1). Applying Cox proportional hazards modeling, shorter tenure was more likely among enrollees with incomes below 150% of the FPL (HR, 1.197; 95% CI, 1.194-1.201) compared with those with incomes of 200% to 400% of the FPL, among Black (HR, 1.379; 95% CI, 1.372-1.387) and Latino (HR, 1.104; 95% CI 1.101-1.107) enrollees compared with White enrollees, and among enrollees who chose high-deductible minimum coverage (HR, 1.437; 95% CI, 1.427-1.447) or bronze plans (HR, 1.119; 95% CI, 1.117-1.122) compared with those in silver plans. Longer tenure was more likely among those who reported self-employment income (HR, 0.556; 95% CI, 0.556-0.558) and those paying a net premium of only $1.00 per member per month (HR, 0.589; 95% CI, 0.587-0.591). During the study period, the lowest premium allowed in California (net of tax credit) was $1.00 per member per month.

### Sources of Coverage Before Enrolling and After Leaving the Marketplace

**Figure 2.**
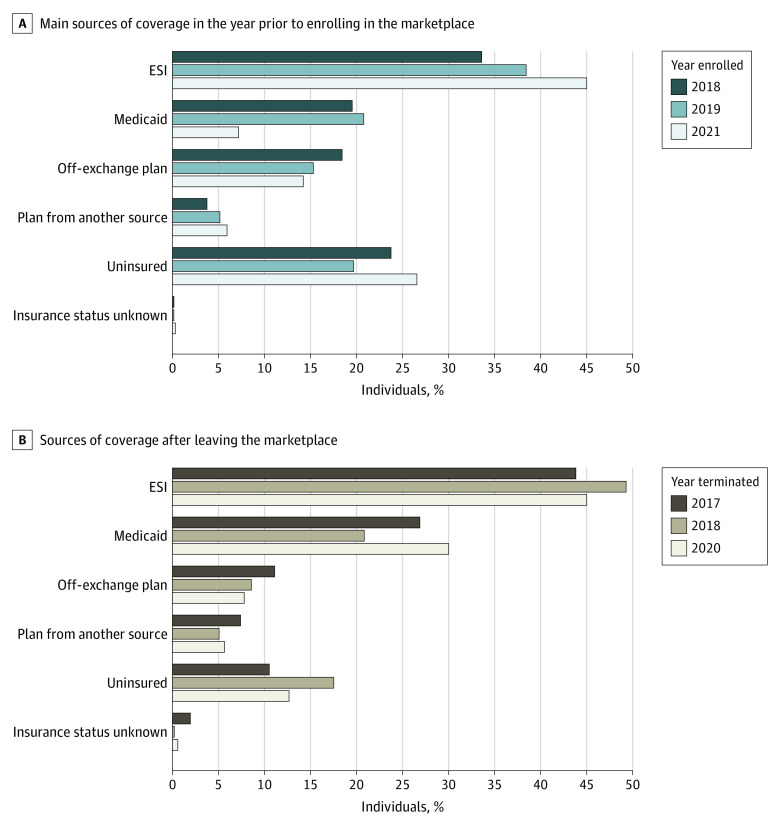
Sources of Health Care Coverage Before Enrolling and After Leaving the Marketplace, by Year A, Prior coverage source was self-reported main coverage source for the year prior to enrolling in the marketplace. Only those who reported not having coverage for the entire year were recorded as uninsured. B, Coverage source was self-reported current coverage source at the time of the survey (during first quarter of the year immediately following open enrollment). ESI indicates employer-sponsored insurance.

Survey data included responses from 6474 terminating individuals across the 3 survey years (2018, 2019, and 2021) and 3097 newly enrolled individuals. Survey results showed that a mean (SE) of 86% (SE, 0.007; 95% CI, 85%-88%) of terminating members acquired coverage through other sources after leaving the marketplaces (Figure 2B). The primary sources of coverage reported were consistent with churn being associated primarily with changing eligibility; a mean (SE) of 46% (SE, 0.011; 95% CI, 44%-48%) of terminating members reported having ESI after leaving the marketplace, and a mean (SE) of 24% (SE, 0.010; 95% CI, 22%-26%) reported enrolling in California’s Medicaid program, Medi-Cal. Assessment of prior sources of coverage among new enrollees similarly suggest that churn was driven by changes in eligibility; a mean (SE) of 56% (0.016) of new members reported either having ESI or Medicaid prior to enrolling in the marketplace (Figure 2A).

A mean (SE) of 14% (SE, 0.007; 95% CI, 12%-15%) of individuals terminating coverage reported being uninsured after open enrollment of the following year, representing a substantially smaller share than previously reported in the federal marketplaces in 2015 (51%).^8^ The share of those terminating coverage who reported being uninsured was highest after the 2018 coverage year, possibly due to the elimination of the ACA’s individual mandate penalty beginning in 2019. When narrowing the sample to exclude “penalty-motivated” (based on responses to various survey items about the penalty), the uninsured rate among 2018 terminating members fell to 13% (95% CI, 11%-16%), consistent with rates of uninsurance among terminating members in other years. Individuals were considered to be penalty motivated if they were either aware of the penalty mandate repeal and said they would have purchased insurance had the mandate been in place or unaware of the penalty mandate repeal and said they would have not purchased insurance without the mandate in place.

Compared with individuals with ESI or Medicaid, 2018 terminating members were more likely to be Latino (40%; 95% CI, 33%-47%) and report not having a college degree (71%; 95% CI, 64%-77%) (Table 2). Individuals who had ESI were more likely to have a bronze plan with the marketplace (43%; 95% CI, 39%-48%). Demographic characteristics of the 2017 and 2020 terminating members are provided in eTables 7 through 9 in Supplement 1.

**Table 2.** Sociodemographic Characteristics of 2018 Terminating Members, by Current Source of Coverage

	Terminating members, weighted point estimate, % (95% CI)			
	All (n = 4517)	Uninsured (n = 875)	With ESI (n = 1674)	With Medi-Cal (n = 874)
Age, y				
18-29	23 (20-26)	23 (17-30)	25 (21-29)	24 (19-31)
30-44	35 (33-38)	30 (23-36)	42 (38-47)	30 (24-36)
45-64	42 (39-45)	48 (20-55)	33 (29-37)	46 (40-53)
Race and ethnicity				
Asian/Pacific Islander	15 (13-17)	13 (9-18)	19 (16-23)	11 (8-15)
Black	3 (2-4)	3 (2-5)	2 (2-4)	4 (3-7)
Latino	23 (20-25)	40 (33-47)	18 (15-22)	25 (19-31)
White	31 (28-34)	24 (19-30)	32 (28-36)	33 (27-40)
Other^a^	9 (7-11)	6 (3-10)	7 (5-10)	15 (10-21)
No response	19 (17-22)	15 (10-20)	21 (18-25)	12 (8-18)
Income group, % of FPL				
<200	47 (44-50)	53 (46-60)	37 (33-42)	73 (66-78)
200-400	37 (34-40)	36 (29-43)	43 (39-47)	23 (18-29)
>400 or unsubsidized	16 (14-18)	11 (7-17)	20 (17-23)	4 (2-7)
Education				
No college degree	44 (41-47)	71 (64-77)	31 (27-35)	53 (46-60)
College degree	56 (53-59)	29 (23-36)	69 (65-73)	47 (40-54)
Region				
Los Angeles	27 (24-30)	30 (24-37)	29 (25-34)	21 (16-27)
Southern California	34 (31-37)	37 (31-45)	26 (22-30)	40 (34-47)
Bay Area	21 (19-24)	11 (8-16)	26 (23-30)	22 (17-28)
Northern California	18 (16-20)	21 (16-27)	18 (15-22)	17 (12-23)
Metal tier^b^				
Bronze or catastrophic	35 (33-38)	35 (29-42)	43 (39-48)	23 (18-29)
Silver 70 or 73	15 (13-17)	15 (10-21)	14 (11-17)	7 (4-12)
Silver 87 or 94	34 (32-37)	34 (32-46)	25 (21-29)	57 (50-63)
Gold or platinum	16 (14-18)	16 (7-17)	18 (15-22)	13 (9-19)

Abbreviations: ESI, employer-sponsored insurance; FPL, federal poverty level; Medi-Cal, California’s Medicaid program.

^a^ Other included individuals who reported their race as American Indian/Alaskan Native, multiple races, and other.

^b^ Metal tier refers to level of benefits provided by a plan, according to actuarial value: a bronze plan covers, on average, 60% of annual costs; a silver plan covers 70%, 73%, 87%, or 94% of costs (participation is restricted according to income level); a gold plan covers 80% of costs; and a platinum plan covers 90% of costs.^11^

### Individuals Terminating Coverage Who Went Uninsured

Among subsidized 2019 renewal candidates (n = 1621), who were enrolled in marketplace coverage through the end of the year and did not report coverage outside of Covered California the following year, only 1.3% (95% CI, 1.2%-1.5%) went uninsured. Table 3 shows variation in the average marginal effects of a consumer’s likelihood to terminate and go uninsured, all else being constant. Latino renewal candidates were 1.5 percentage points (95% CI, 0.8-2.3 percentage points) more likely to go uninsured than White renewal candidates, and those who were aware of the individual mandate repeal were 1.7 percentage points (95% CI, 1.0-2.4 percentage points) more likely to go uninsured than those who were unaware. Renewal candidates with no expected physician visits in the coming year were 4.8 percentage points (95% CI, 2.4-7.2 percentage points) more likely to go uninsured, and those who rated the value of their plan as low were 2.0 percentage points (95% CI, 0.5-3.4 percentage points) more likely to do so. Expected health care use appeared to be a dominant factor in consumers’ decision-making process. Among those who rated their plan as poor, consumers without any expected use were 10.6 percentage points (95% CI, 5.3-15.8 percentage points) more likely to go uninsured; even among those who gave their plans higher ratings, consumers without any expected use were 4.3 percentage points (95% CI, 2.1-6.6 percentage points) more likely to go uninsured.

**Table 3.** Marginal Effects of Decision to Renew Coverage or Terminate and Go Uninsured^a^

	Share of participants uninsured, % (SE)	Average marginal effect	
		Effect size (SE) [95% CI]	*P* value
All subsidized renewal candidates	1.3 (0.001)	NA	NA
Age, y			
18-29	1.7 (0.003)	0.003 (0.004) [−0.006 to 0.011]	.54
30-44	1.4 (0.002)	−0.001 (0.003) [−0.007 to 0.005]	.76
45-64	1.2 (0.001)	1 [Reference]	NA
Race or ethnicity			
Asian/Pacific Islander	1.0 (0.002)	0.002 (0.002) [−0.003 to 0.067]	.46
Black	0.9 (0.003)	0.006 (0.005) [−0.005 to 0.017]	.27
Latino	2.6 (0.003)	0.015 (0.004) [0.008 to 0.023]	<.001
White	0.8 (0.001)	1 [Reference]	NA
Other^b^	0.9 (0.003)	0.005 (0.005) [−0.006 to 0.015]	.40
No response	2.3 (0.015)	0.021 (0.016) [−0.011 to 0.053]	.20
Awareness of penalty repeal			
Aware	2.4 (0.001)	0.017 (0.003) [0.010 to 0.024]	<.001
Unaware	0.7 (0.003)	1 [Reference]	NA
Change in net premium, %	NA	−0.011 (0.021) [−0.053 to 0.030]	.59
Physician visits expected, No.			
0	5.0 (0.009)	0.048 (0.012) [0.024 to 0.072]	<.001
≥1	1.0 (0.001)	1 [Reference]	NA
Insurance status in 2018			
Insured entire year	1.1 (0.001)	1 [Reference]	NA
Uninsured some of year	3.2 (0.005)	0.016 (0.008) [0.004 to 0.028]	.008
Participant rating of health plan value			
Excellent, good, fair	1.1 (0.001)	1 [Reference]	NA
Poor	3.9 (0.008)	0.020 (0.005 to 0.034]	.009
Metal tier^c^			
Bronze	1.7 (0.002)	−0.004 (0.004) [−0.013 to 0.005]	.36
Silver	1.2 (0.001)	0.000 (0.004) [−0.009 to 0.008]	.95
Gold or platinum	1.0 (0.002)	1 [Reference]	NA
Income group, % of FPL			
≤200	1.3 (0.001)	1 [Reference]	NA
>200-400	1.2 (0.002)	0.003 (0.003) [−0.003 to 0.009]	.31
>400 or unsubsidized	1.8 (0.004)	0.015 (0.006) [0.003 to 0.028]	.01
Education			
College degree	1.0 (0.001)	1 [Reference]	NA
No college degree	1.5 (0.001)	0.006 (0.003) [0.001 to 0.011]	.02
Location			
Los Angeles	1.5 (0.002)	0.008 (0.004) [0.000 to 0.015]	.04
Southern California	1.7 (0.002)	0.007 (0.003) [0.002 to 0.013]	.01
Bay Area	0.8 (0.002)	0.002 (0.003) [−0.004 to 0.009]	.50
Northern California	1.1 (0.002)	1 [Reference]	NA
Expected use over value^d^			
No visits	NA	NA	NA
Excellent, good, or fair	4.2 (0.009)	0.043 (0.012) [0.021 to 0.066]	<.001
Poor	7.9 (0.029)	0.106 (0.027) [0.053 to 0.158]	<.001

Abbreviations: FPL, federal poverty level; NA, not applicable.

^a^ Authors’ analysis of California Health Coverage survey data supplemented with Covered California administrative data (n = 1621).

^b^ Other included individuals who reported their race as American Indian/Alaskan Native, multiple races, or other.

^c^ Metal tier refers to level of benefits provided by a plan, according to actuarial value: a bronze plan covers, on average, 60% of annual costs; a silver plan covers 70%, 73%, 87%, or 94% of costs (participation is restricted according to income level); a gold plan covers 80% of costs; and a platinum plan covers 90% of costs.^11^

^d^ These rows refer to the interaction of expected use of care and perceived plan value (ie, the perceived plan value among people who reported no expected use of care compared with that among those who did expect to use care).

## Discussion

Within a fragmented health care coverage landscape, ACA marketplaces offer the opportunity for individuals to maintain coverage in the face of employment or life circumstance shocks. This cross-sectional analysis of tenure suggests that marketplaces served both long-term enrollees, who often stayed for 2 or more years, and shorter-term enrollees, many of whom enrolled for less than 1 year. Long-term enrollees were more likely to report self-employment income, indicating a lack of ESI, and were more likely be enrolled in plans with low net premiums. Shorter-term enrollees, however, were more likely to have low incomes, be Black or Latino, and be enrolled in high-deductible plans. Shorter enrollment periods should not necessarily be viewed as problematic because linked survey data suggest that many of these consumers may experience changes that affect their eligibility for Medicaid or receive offers of insurance through employers. These 2 eligibility changes alone accounted for 70% of Covered California’s terminations and 56% of new enrollments, confirming early expectations for how marketplaces could mitigate uninsurance amid eligibility churn.^12^

However, marketplace churn is not entirely explained by coverage transitions, with 14% of terminating members going uninsured (showing substantial demographic variation from those who stay covered). Our analysis of renewal candidates allowed us to better understand the motivations behind the decision to go uninsured and suggests that perceived low plan value and low expected use, and not cost, play a greater role in decisions to disenroll from coverage. How consumers experience their plan matters; those who do not regularly access care are less likely to pay for the protective value of insurance.

The COVID-19 pandemic reinforced the importance of insurance marketplaces as a home for people experiencing coverage disruptions.^13^ Amid widespread unemployment and work reductions, Covered California and HealthCare.gov reported record enrollment during the 2020 special enrollment period.^14,15^ In response to the disruptions, California and 11 other state-based marketplaces created an emergency special enrollment period to allow anyone impacted by the pandemic to take up marketplace coverage.

The US Congress also addressed the demand for coverage by enacting new, temporary measures through the American Rescue Plan Act of 2021 and Inflation Reduction Act of 2022 to improve marketplace affordability, increasing subsidies for those already eligible and extending new subsidies to many middle-income consumers. The new financial help provided a refreshed opportunity for the remaining uninsured individuals who might previously have found that marketplace plans were unaffordable. Initial data from the Centers for Medicare & Medicaid Services indicate that these steps helped to reduce churn, preventing many from being uninsured, and even point toward further reductions in the uninsured rate.^16^ To further reduce the number of uninsured during such shocks, policy makers could also consider factors other than premium price—such as high out-of-pocket costs—that influence decisions about whether to take up coverage.

Having successfully implemented tools to minimize coverage transitions during a pandemic, policy makers should consider further options that smooth churn from ESI and Medicaid, which our data found occurred in large volumes even outside of coverage shocks. Changes under the public health emergency declared by the U.S Department of Health and Human Services on January 31, 2020, reduced churn from Medicaid to a fraction of levels in a normal year, but as traditional eligibility rules resume, marketplaces will need to use additional tools to prevent gaps in coverage.^17^ Autoenrollment between the marketplaces and Medicaid presents an opportunity for further coordination between the 2 programs, given that many individuals regularly experience changes in income that shifts their eligibility between the 2 subsidized enrollment options.

California has already taken steps to ease these transitions; beginning in 2022, state legislation requires automatic plan selection of consumers transitioning from Medicaid into marketplace coverage, in an effort to remove non–price-related barriers to enrollment.^18^ Additionally, this legislation mandates outreach by the marketplace to individuals who are losing other commercial coverage, in an effort to smooth transitions from ESI. Congressional action in the American Rescue Plan Act made marketplace coverage effectively free in 2021 for those who are receiving unemployment insurance, acknowledging the importance of job loss in health care coverage and showing potential for other facilitated enrollment strategies if unemployment insurance recipients report loss of job-based coverage.

Facilitated enrollment policies acknowledge a key feature of marketplaces and churn: many individuals experiencing coverage transitions may not be fully aware of the available coverage options. In addition to policies that improve affordability and facilitate enrollment, investments in outreach should also continue to further reduce the long-term uninsured, many of whom are estimated to be eligible for marketplace subsidies.^19^

### Limitations

This study has limitations, including its focus on the marketplace experience in 1 state. Churn dynamics and their implications will vary in other settings, depending on factors such as the prevalence of employer coverage, the status of the Medicaid expansion, and state policies on promoting coverage. Although our examination of churn was an attempt to understand the marketplace’s role in a broader health care landscape, we only assessed churn for individuals who had taken up nongroup coverage.

A second limitation concerns the timing of the self-reported sources of coverage. Because the surveys reported current coverage in the spring, the results might not be fully representative of postmarketplace coverage for enrollees who leave the exchange in the summer or early fall. Similarly, the survey asked respondents for their main source of coverage in the year prior to enrolling and may therefore not represent their coverage experience immediately prior to their marketplace coverage.

A third limitation concerns the survival model. Enrollment and termination of coverage on the exchange may involve both time-varying characteristics and competing risks, each of which could bias survival modeling results. Unfortunately, the data available for this study did not allow us to identify these characteristics.

## Conclusions

In this cross-sectional study, we found that most ACA marketplace churn in California was the result of changes in eligibility, given that most individuals reported Medicaid or employer coverage either before beginning or after terminating their marketplace coverage. Administrative data revealed that churn was a core dynamic within the marketplaces, rather than a sign of instability, and suggests that it is likely to continue. Marketplace policies such as ongoing outreach and streamlined coverage transitions through facilitated enrollment will be integral to continue reducing the number of uninsured and mitigating the effects of coverage disruptions.

## Notes

Supplement. **eAppendix 1.** Survival Analysis **eTable 1.** Variables Used for Survival Analysis **eFigure 1.** Survival Curve of Coverage by Month Using Kaplan-Meier Estimator **eTable 2.** Relative Risk of Coverage Terminations (Alternate Models) **eAppendix 2.** Details on the California Health Coverage Survey **eTable 3.** California Health Coverage Survey Response Rates by Consumer Cohort **eTable 4.** Sample Distribution on Selected Database Demographic Variables Among 2017 Terminating Members **eTable 5.** Sample Distribution on Selected Database Demographic Variables Among 2018 Terminating Members **eTable 6.** Sample Distribution on Selected Database Demographic Variables Among 2020 Terminating Members **eTable 7.** Sociodemographic Characteristics of 2017 Terminating Members, by Current Source of Coverage **eTable 8.** Sociodemographic Characteristics of 2018 Terminating Members, by Current Source of Coverage **eTable 9.** Sociodemographic Characteristics of 2020 Terminating Members, by Current Source of Coverage **eAppendix 3.** Modeling Terminations Who Go Uninsured **eFigure 2.** Self-Reported Reasons for Terminating Coverage, Among Uninsured Renewal Candidates **eTable 10.** Observations of Independent Variables Among Subsidized Renewal Candidates **eTable 11.** Logistical Regression Modeling Output: Decision to Renew or Terminate and Go Uninsured **eTable 12.** Average Marginal Effects of Decision to Renew or Terminate and Go Uninsured (Using Gross Premium Rate Changes Instead of Net-of-Subsidy Rate Changes) **eTable 13.** Average Marginal Effects of Decision to Renew or Terminate and Go Uninsured (Alternative Specification Among Subsidized Renewal Candidates With Updated Eligibility During 2019 Open Enrollment)

Supplement 2.
Data Sharing Statement
