# Supplementary material for: Assessment of Churn in Coverage Among California’s Health Insurance Marketplace Enrollees
Source: JAMA Health Forum. 2022 Dec 2;3(12):e224484. doi: 10.1001/jamahealthforum.2022.4484 (PMC9719048; doi:10.1001/jamahealthforum.2022.4484)
Supplement: Supplement. — eAppendix 1. Survival Analysis eTable 1. Variables Used for Survival Analysis eFigure 1. Survival Curve of Coverage by Month Using Kaplan-Meier Estimator eTable 2. Relative Risk of Coverage Terminations (Alternate Models) eAppendix 2. Details on the California Health Coverage Survey eTable 3. California Health Coverage Survey Response Rates by Consumer Cohort eTable 4. Sample Distribution on Selected Database Demographic Variables Among 2017 Terminating Members eTable 5. Sample Distribution on Selected Database Demographic Variables Among 2018 Terminating Members eTable 6. Sample Distribution on Selected Database Demographic Variables Among 2020 Terminating Members eTable 7. Sociodemographic Characteristics of 2017 Terminating Members, by Current Source of Coverage eTable 8. Sociodemographic Characteristics of 2018 Terminating Members, by Current Source of Coverage eTable 9. Sociodemographic Characteristics of 2020 Terminating Members, by Current Source of Coverage eAppendix 3. Modeling Terminations Who Go Uninsured eFigure 2. Self-Reported Reasons for Terminating Coverage, Among Uninsured Renewal Candidates eTable 10. Observations of Independent Variables Among Subsidized Renewal Candidates eTable 11. Logistical Regression Modeling Output: Decision to Renew or Terminate and Go Uninsured eTable 12. Average Marginal Effects of Decision to Renew or Terminate and Go Uninsured (Using Gross Premium Rate Changes Instead of Net-of-Subsidy Rate Changes) eTable 13. Average Marginal Effects of Decision to Renew or Terminate and Go Uninsured (Alternative Specification Among Subsidized Renewal Candidates With Updated Eligibility During 2019 Open Enrollment) [file jamahealthforum-e224484-s001.pdf]

Supplemental Online Content

Wolf E, Slosar M, Menashe I. Assessment of churn in coverage among California's health insurance marketplace enrollees. 2022;3(12):e224484. doi:10.1001/jamahealthforum.2022.4484

eAppendix 1. Survival Analysis

eTable 1. Variables Used for Survival Analysis

eFigure 1. Survival Curve of Coverage by Month Using Kaplan-Meier Estimator

eTable 2. Relative Risk of Coverage Terminations (Alternate Models)

eAppendix 2. Details on the California Health Coverage Survey

eTable 3. California Health Coverage Survey Response Rates by Consumer Cohort

eTable 4. Sample Distribution on Selected Database Demographic Variables Among 2017 Terminating Members

eTable 5. Sample Distribution on Selected Database Demographic Variables Among 2018 Terminating Members

eTable 6. Sample Distribution on Selected Database Demographic Variables Among 2020 Terminating Members

eTable 7. Sociodemographic Characteristics of 2017 Terminating Members, by Current Source of Coverage

eTable 8. Sociodemographic Characteristics of 2018 Terminating Members, by Current Source of Coverage

eTable 9. Sociodemographic Characteristics of 2020 Terminating Members, by Current Source of Coverage

eAppendix 3. Modeling Terminations Who Go Uninsured

eFigure 2. Self-Reported Reasons for Terminating Coverage, Among Uninsured Renewal Candidates

eTable 10. Observations of Independent Variables Among Subsidized Renewal Candidates

eTable 11. Logistical Regression Modeling Output: Decision to Renew or Terminate and Go Uninsured Among Subsidized Renewal Candidates

eTable 12. Average Marginal Effects of Decision to Renew or Terminate and Go Uninsured (Alternative Specification Using Gross Premium Rate Changes Instead of Net-of-Subsidy Rate Changes)

© 2022 Wolf E et al. *JAMA Network Open.*

eTable 13. Average Marginal Effects of Decision to Renew or Terminate and Go Uninsured (Alternative Specification Among Subsidized Renewal Candidates With Updated Eligibility During 2019 Open Enrollment)

eReferences

This supplemental material has been provided by the authors to give readers additional information about their work.

© 2022 Wolf E et al. *JAMA Network Open.*

eAppendix 1. Survival Analysis

This study seeks to shed light on the high rate of turnover in the individual market by assessing both the characteristics of short- and longer-term enrollees, as well as documenting the sources of coverage reported by enrollees before entering and after leaving the marketplace. Theoretically, all consumers will eventually drop coverage at some point, either because they have another source of coverage, or are no longer able to maintain coverage (e.g., due to cost, moving, or perhaps in some cases, death). In our main analysis, we focus on descriptive measures of median enrollment and estimate hazard ratios using a Cox proportional hazard model to better understand the enrollee and marketplace experience characteristics associated with shorter or longer spells of enrollment.

Data and Modeling Approach

For both the descriptive and multi-variate analyses, we use a dataset with one record per effectuated coverage segment, defined as an uninterrupted month of continuous coverage. The data include individual, demographic, plan, and eligibility characteristics (all categorical) covariates, as detailed in the table below. Note that the extraction of data from Covered California defined a continuous coverage segment in a way that collapsed what may sometimes include multiple plan selection or eligibility events for a single person (e.g., renewals for new plan contract years, changes between issuer or tier, and changes in premium due to shifts in income) into a single summary of the uninterrupted coverage duration.

We selected covariates by creating a list of attributes that could plausibly impact consumer decision to retain coverage that were known to be available in the Covered California data. We then used a combination of visual representations of the Kaplan-Meier survival function and log-rank tests to assess each covariate for inclusion. Several dimensions showed some “crossing” between some of the categorical values, but we elected to retain these covariates in the final model based on strong theoretical grounds and the relatively mild overlap of otherwise quite similar lines that emerged.[1] We also reviewed the covariance matrix after model run to check for high levels of correlation between covariates.

Nearly all of the variables originally created by Covered California were used. In the case of income, we sought to use the FPL bracket at the time of initial plan selection. Where missing, we instead used the latest known FPL for the enrollment. Additionally, because federal subsidies for insurance increase as income decreases – and this is the primary impact of income we are interested in for this setting – the category for income groups consumers who are not eligible for tax credits as Unsubsidized, regardless of whether they reported income. As a sensitivity check on this method of grouping by income and eligibility, we also ran models for those eligible and those not eligible separately (see Models 2 and 3 in eTable 2 below). We also ran alternative models in which the FPL bracket was interacted with a dummy for subsidy eligibility (not shown).

Additionally, four covariates originally requested were explored but not included in the final model due to uncertainty relayed from Covered California about the data quality for these elements in the data used for this study: a flag for having ever been found conditionally eligible for Advanced Premium Tax Credits (APTC), a flag for having reported Unemployment Income, and flags for having transitioned between Covered California and the state Medicaid agency.

---

[1] The overlapping curves included: a) Income: some more rapid reduction in failure among the 400%+ FPL group. This could have been due to program changes in eligibility for subsidies with this group towards the end of the study period; b) Age: a very slight crossing of close and nearly aligned curves for younger (0-18) and middle aged (30-44) enrollees; c) Race/Ethnicity: survival curves for white and “Other” were nearly aligned, and showed slight crossing; d) Metal Tier: the gold/platinum curve crossed other tiers slightly, becoming more likely to survive over time relative to the other tiers.

eTable 1. Variables Used for Survival Analysis

Variable	Role in model	Description
cov_termed	event	Dummy variable denoting whether the coverage ended by December 31, 2021 (failure, denoted as a 1) or was censored (0)
months_to_2021	time	Time unit – months of coverage
fpl_grp_n_4	covariate	Income as percentage of federal poverty at time of plan selection. Consumers who are not eligible for tax credits all coded as Unsubsidized regardless of whether they reported income.
age_bracket_sum_n	covariate	Age at time of plan selection
race_ethnicity_grp_n	covariate	Race/ethnicity
language_spkn_grp_n	covariate	Spoken language preference as of time of initial plan selection
gender_n	covariate	Gender
hh_size_4	covariate	Household size (with 4 or more grouped in a single category)
rating_regions	covariate	Geographic region of the state used for plan pricing, using a roll-up of the state's 19 rating regions
metal_tier_sum_n	covariate	Metal tier of plan choosing at initial plan selection
broker	covariate	Whether insurance broker assisted with initial plan selection
sep	covariate	Whether initial plan selection occurred during a special enrollment period (after the close of the open enrollment period)
net_premium_1_or_less	covariate	Whether the consumer's final net premium was $1 or less as of the time of termination.
ever_saws	covariate	Whether the consumer was ever part of a household that had interaction with the county Medicaid system during the coverage period
self_empl_incm_flg	covariate	Whether the consumer reported any self-employment income information at the time of plan selection
x7	covariate in alternate specification	Dummy indicating being ineligible for subsidies (x7=1)

Assumptions and Limitations

Many characteristics of theoretical interest for analysis of tenure – such as unobserved attitudes towards coverage, propensity to need or use health care, financial hardship, and others – were not available in the administrative data for this study and are thus not included in the analysis of factors associated with tenure and disenrollment.

Additionally, for the survival analysis, the decision to drop coverage to go uninsured or to obtain other sources of coverage may well be considered a "competing risks" setting, as consumers could also drop coverage due to moving, death, or other factors. The literature on survival models emphasizes the importance of accounting for competing risks to avoid overstating the risk from the non-competing failure event (e.g. through the use of cumulative incidence function instead of the Kaplan-Meier estimator or Cox proportional hazard models).[1] However, in the administrative data from Covered California that is used for the survival analysis, we only observe that a consumer's coverage ends, and thus cannot separately identify the competing risks in the data. As a result, we consider the proportional hazards analysis here to be an assessment of the risk of terminating coverage, but not necessarily the risk of a specific reason or set of reasons to terminate coverage. As a robustness check on the idea of competing risks modeling, we exploit one key reason for leading coverage for which we have a very good proxy in

© 2022 Wolf E et al. *JAMA Network Open.*

the data: that the vast majority of consumers who turn 65 will exist to Medicare. We run a competing risks regression using *stcrreg* in Stata 17, following Fine and Grey (1999), using the same set of covariates as the main model, but specifying being over 64 at the time enrollment ends as a competing risk (see Model 5 in eTable 2 below). [2] For performance reasons, the competing risk regression was run on a random sample of the overall data.

After running the hazard models, we reviewed plots of the Cox-Snell residuals to assess overall model fit, as well as Schoenfeld residuals with a lowess smoother to visually inspect each covariate for possible violations of the proportional hazard assumptions.

However, we note that the construction of the data provided by Covered California made important assumptions by fixing enrollment features that are known to vary over time (in particular, income, premium amounts, and metal tier of plan enrollment). Future work in this area – provided more detailed data are available – could better explore the time-varying aspects of some of the covariates.

Additionally, for simplicity, we also treat repeated enrollment segments by the same individual – provided it is separated by at least a one-month gap in coverage – as two distinct "enrollees." Repeated event analysis is beyond the scope of this study, but we provide a sensitivity check on this approach by running the hazards model limited to only the first enrollment segment for each individual (shown in Model 4 of eTable 2 below).

Results

To complement the median time to disenrollment from the main analysis, we provide the visual representation the churn of enrollees from Covered California using a Kaplan-Meier curve in eFigure 2 below, which shows that half of enrollees are estimated to end their enrollment with Covered California by the 14th month.

For the proportional hazard models, we find substantively similar results in Model 2 (subsidy eligible only), Model 3 (subsidy ineligible only) and Model 4 (first enrollment per individual). For Model 5 the competing risks regression – we see substantively similar results for all covariates except age, where we see the hazard ratio for the younger enrollees is larger in the competing risks regression (as the exits to Medicare are no longer considered "failure" under this approach).

The review of the correlations among covariates did not cause us to alter model specifications at all, and none of the covariate residual plots suggested violations of the proportional hazard assumption.

© 2022 Wolf E et al. *JAMA Network Open.*

eFigure 1. Survival Curve of Coverage by Month Using Kaplan-Meier Estimator

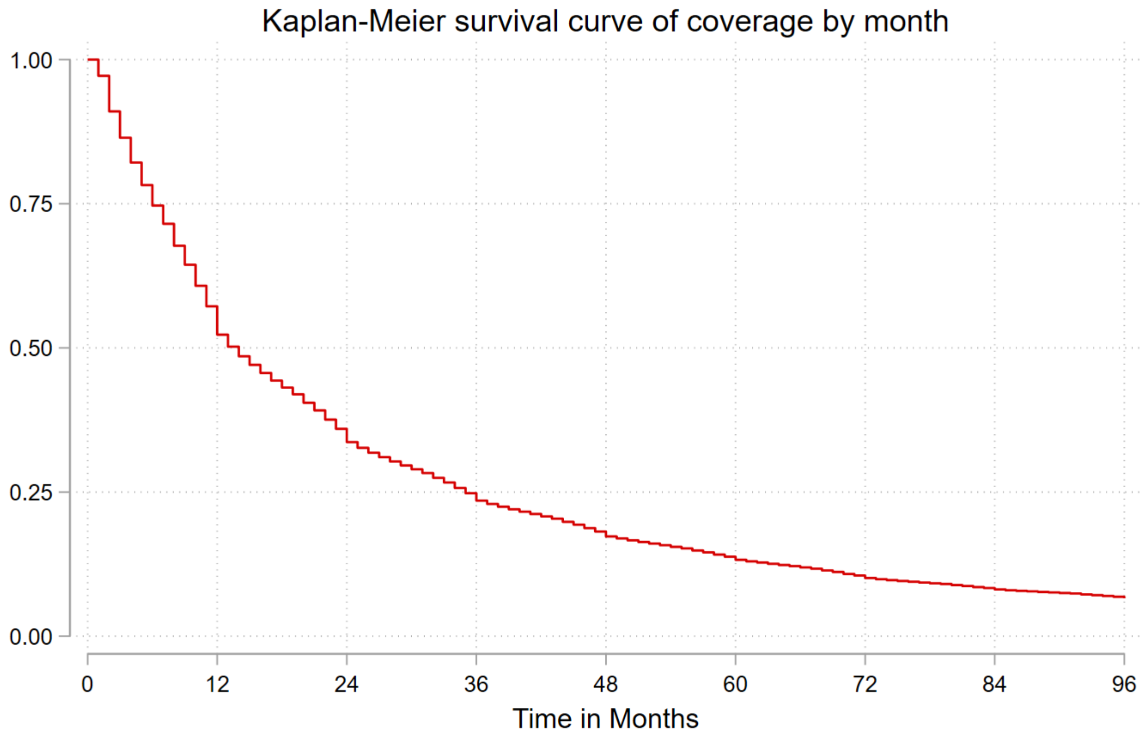

© 2022 Wolf E et al. *JAMA Network Open.*

**eTable 2. Relative Risk of Coverage Terminations (alternate models) – Panel A**				
Model 1:<br>Main Specification	**Relative Risk of Coverage Termination**<br>(Cox Proportional Hazard Ratios)			
	**Hazard Ratio**	se	95% Confidence Interval	p-value
**Income Group** (as percent of Federal Poverty Level)				
150% FPL or less	**1.198**	(134.73)	[1.195,1.201]	(0.000)
150% FPL to 200% FPL	**1.017**	(14.73)	[1.015,1.020]	(0.000)
200% FPL to 400% FPL	**1**	(.)	[1,1]	(.)
400% FPL or greater or unsubsidized	**1.038**	(20.49)	[1.034,1.042]	(0.000)
**Age Group**				
Age 0 to 17	**1.197**	(96.27)	[1.193,1.202]	(0.000)
Age 18 to 29	**1.287**	(205.85)	[1.284,1.290]	(0.000)
Age 30 to 44	**1.230**	(180.29)	[1.227,1.233]	(0.000)
Age 45 to 64	**1**	(.)	[1,1]	(.)
**Race / Ethnicity**				
(nonrespondent)	**1.069**	(48.85)	[1.066,1.072]	(0.000)
Asian American, Native Hawaiian or Other Pacific Islander	**0.967**	(-21.10)	[0.964,0.970]	(0.000)
Black or African American	**1.379**	(112.44)	[1.372,1.387]	(0.000)
Latino	**1.104**	(69.89)	[1.101,1.107]	(0.000)
Other	**1.008**	(4.23)	[1.004,1.012]	(0.000)
White	**1**	(.)	[1,1]	(.)
**Language Spoken**				
Asian or Pacific Islander languages	**0.868**	(-61.76)	[0.864,0.872]	(0.000)
English	**1**	(.)	[1,1]	(.)
Other	**1.183**	(67.06)	[1.177,1.189]	(0.000)
Spanish	**0.939**	(-35.77)	[0.936,0.942]	(0.000)
**Health Plan Metal Tier**				
Minimum Coverage	**1.437**	(104.29)	[1.427,1.447]	(0.000)
Bronze	**1.119**	(101.65)	[1.117,1.122]	(0.000)
Silver	**1**	(.)	[1,1]	(.)
Gold / Platinum	**0.976**	(-16.03)	[0.973,0.979]	(0.000)
**Additional Factors**				
Broker Assistance	**0.865**	(-149.81)	[0.863,0.867]	(0.000)
Special enrollment period	**1.192**	(174.22)	[1.190,1.195]	(0.000)
Monthly premium $1 or less	**0.589**	(-322.91)	[0.587,0.591]	(0.000)
Household w/ Medicaid interaction	**0.758**	(-290.01)	[0.756,0.759]	(0.000)
Self-employment income	**0.557**	(-474.38)	[0.556,0.558]	(0.000)
**Gender**				
Female	**1**	(.)	[1,1]	(.)
Male	**0.998**	(-2.58)	[0.996,0.999]	(0.010)
**Household Size**				
No household size information	**0.961**	(-15.13)	[0.957,0.966]	(0.000)
1	**1**	(.)	[1,1]	(.)
2	**1.050**	(33.29)	[1.047,1.053]	(0.000)
3	**1.064**	(37.64)	[1.061,1.068]	(0.000)
4 or more	**1.037**	(29.87)	[1.035,1.040]	(0.000)
**Rating Region**				
Northern California	**1.015**	(11.15)	[1.012,1.017]	(0.000)
Bay Area	**1.040**	(29.51)	[1.037,1.043]	(0.000)
Los Angeles	**1**	(.)	[1,1]	(.)
Southern California	**1.058**	(47.33)	[1.056,1.061]	(0.000)
**N**	**6,521,763**			

© 2022 Wolf E et al. *JAMA Network Open.*

**eTable 2. Relative Risk of Coverage Terminations (alternate models) – Panel B**				
Model 2:<br>Subsidy-eligible only	**Relative Risk of Coverage Termination**<br>(Cox Proportional Hazard Ratios)			
	**Hazard Ratio**	se	95% Confidence Interval	p-value
**Income Group** (as percent of Federal Poverty Level)				
150% FPL or less	**1.197**	(129.72)	[1.194,1.201]	(0.000)
150% FPL to 200% FPL	**1.020**	(16.40)	[1.018,1.022]	(0.000)
200% FPL to 400% FPL	**1**	(.)	[1,1]	(.)
400% FPL or greater or unsubsidized	**1.158**	(49.55)	[1.152,1.165]	(0.000)
**Age Group**				
Age 0 to 17	**1.211**	(87.42)	[1.206,1.216]	(0.000)
Age 18 to 29	**1.295**	(199.39)	[1.291,1.298]	(0.000)
Age 30 to 44	**1.229**	(167.98)	[1.226,1.232]	(0.000)
Age 45 to 64	**1**	(.)	[1,1]	(.)
**Race / Ethnicity**				
(nonrespondent)	**1.072**	(46.98)	[1.069,1.075]	(0.000)
Asian American, Native Hawaiian or Other Pacific Islander	**0.958**	(-24.80)	[0.955,0.962]	(0.000)
Black or African American	**1.385**	(106.48)	[1.376,1.393]	(0.000)
Latino	**1.102**	(64.28)	[1.099,1.105]	(0.000)
Other	**1.004**	(2.16)	[1.000,1.008]	(0.031)
White	**1**	(.)	[1,1]	(.)
**Language Spoken**				
Asian or Pacific Islander languages	**0.871**	(-58.03)	[0.866,0.875]	(0.000)
English	**1**	(.)	[1,1]	(.)
Other	**1.194**	(64.47)	[1.188,1.201]	(0.000)
Spanish	**0.939**	(-35.09)	[0.935,0.942]	(0.000)
**Health Plan Metal Tier**				
Minimum Coverage	**1.362**	(59.53)	[1.349,1.376]	(0.000)
Bronze	**1.137**	(107.89)	[1.135,1.140]	(0.000)
Silver	**1**	(.)	[1,1]	(.)
Gold / Platinum	**0.974**	(-15.42)	[0.971,0.978]	(0.000)
**Additional Factors**				
Broker Assistance	**0.853**	(-153.66)	[0.852,0.855]	(0.000)
Special enrollment period	**1.173**	(145.35)	[1.170,1.175]	(0.000)
Monthly premium $1 or less	**0.588**	(-318.03)	[0.586,0.590]	(0.000)
Household w/ Medicaid interaction	**0.746**	(-285.42)	[0.744,0.747]	(0.000)
Self-employment income	**0.555**	(-454.79)	[0.553,0.556]	(0.000)
**Gender**				
Female	**1**	(.)	[1,1]	(.)
Male	**1.003**	(3.20)	[1.001,1.005]	(0.001)
**Household Size**				
No household size information	**1.817**	(89.28)	[1.794,1.841]	(0.000)
1	**1**	(.)	[1,1]	(.)
2	**1.050**	(31.72)	[1.047,1.053]	(0.000)
3	**1.065**	(36.55)	[1.062,1.069]	(0.000)
4 or more	**1.043**	(32.99)	[1.041,1.046]	(0.000)
**Rating Region**				
Northern California	**1.011**	(7.63)	[1.008,1.014]	(0.000)
Bay Area	**1.032**	(21.28)	[1.029,1.034]	(0.000)
Los Angeles	**1**	(.)	[1,1]	(.)
Southern California	**1.060**	(45.43)	[1.058,1.063]	(0.000)

© 2022 Wolf E et al. *JAMA Network Open.*

eTable 2. Relative Risk of Coverage Terminations (alternate models) – Panel C				
Model 3:<br>Not subsidy-eligible only	**Relative Risk of Coverage Termination**<br>(Cox Proportional Hazard Ratios)			
	**Hazard Ratio**	se	95% Confidence Interval	p-value
**Income Group** (as percent of Federal Poverty Level)				
150% FPL or less	**1.162**	(22.42)	[1.147,1.178]	(0.000)
150% FPL to 200% FPL	**1.035**	(5.53)	[1.022,1.047]	(0.000)
200% FPL to 400% FPL	**1**	(.)	[1,1]	(.)
400% FPL or greater or unsubsidized	**0.994**	(-1.49)	[0.986,1.002]	(0.136)
**Age Group**				
Age 0 to 17	**1.133**	(32.00)	[1.125,1.142]	(0.000)
Age 18 to 29	**1.212**	(50.12)	[1.203,1.221]	(0.000)
Age 30 to 44	**1.221**	(61.26)	[1.214,1.229]	(0.000)
Age 45 to 64	**1**	(.)	[1,1]	(.)
**Race / Ethnicity**				
(nonrespondent)	**1.046**	(12.52)	[1.039,1.054]	(0.000)
Asian American, Native Hawaiian or Other Pacific Islander	**1.020**	(4.90)	[1.012,1.028]	(0.000)
Black or African American	**1.335**	(35.29)	[1.314,1.357]	(0.000)
Latino	**1.099**	(23.32)	[1.090,1.107]	(0.000)
Other	**1.015**	(3.06)	[1.005,1.025]	(0.002)
White	**1**	(.)	[1,1]	(.)
**Language Spoken**				
Asian or Pacific Islander languages	**0.937**	(-7.18)	[0.920,0.953]	(0.000)
English	**1**	(.)	[1,1]	(.)
Other	**1.149**	(22.42)	[1.135,1.163]	(0.000)
Spanish	**0.970**	(-3.57)	[0.954,0.986]	(0.000)
**Health Plan Metal Tier**				
Minimum Coverage	**1.395**	(62.22)	[1.381,1.410]	(0.000)
Bronze	**0.986**	(-4.48)	[0.981,0.992]	(0.000)
Silver	**1**	(.)	[1,1]	(.)
Gold / Platinum	**0.933**	(-20.30)	[0.926,0.939]	(0.000)
**Additional Factors**				
Broker Assistance	**0.954**	(-16.66)	[0.949,0.959]	(0.000)
Special enrollment period	**1.288**	(97.01)	[1.282,1.295]	(0.000)
Monthly premium $1 or less	**0.575**	(-55.71)	[0.564,0.586]	(0.000)
Household w/ Medicaid interaction	**0.812**	(-79.48)	[0.808,0.816]	(0.000)
Self-employment income	**0.554**	(-145.69)	[0.549,0.558]	(0.000)
**Gender**				
Female	**1**	(.)	[1,1]	(.)
Male	**0.972**	(-11.74)	[0.967,0.976]	(0.000)
**Household Size**				
No household size information	**0.895**	(-28.01)	[0.889,0.902]	(0.000)
1	**1**	(.)	[1,1]	(.)
2	**1.061**	(11.80)	[1.051,1.072]	(0.000)
3	**1.067**	(11.00)	[1.055,1.079]	(0.000)
4 or more	**0.971**	(-7.44)	[0.963,0.978]	(0.000)
**Rating Region**				
Northern California	**1.031**	(7.76)	[1.023,1.039]	(0.000)
Bay Area	**1.073**	(21.15)	[1.066,1.080]	(0.000)
Los Angeles	**1**	(.)	[1,1]	(.)
Southern California	**1.037**	(11.24)	[1.030,1.043]	(0.000)
**N**	**6,521,763**			

© 2022 Wolf E et al. *JAMA Network Open.*

eTable 2. Relative Risk of Coverage Terminations (alternate models) – Panel D				
Model 4: Only one enrollment per individual (first)	**Relative Risk of Coverage Termination** (Cox Proportional Hazard Ratios)			
	**Hazard Ratio**	se	95% Confidence Interval	p-value
**Income Group** (as percent of Federal Poverty Level)				
150% FPL or less	**1.195**	(122.41)	[1.192,1.199]	(0.000)
150% FPL to 200% FPL	**1.016**	(12.40)	[1.013,1.019]	(0.000)
200% FPL to 400% FPL	**1**	(.)	[1,1]	(.)
400% FPL or greater or unsubsidized	**1.036**	(18.16)	[1.032,1.040]	(0.000)
**Age Group**				
Age 0 to 17	**1.199**	(90.90)	[1.195,1.204]	(0.000)
Age 18 to 29	**1.278**	(185.59)	[1.274,1.281]	(0.000)
Age 30 to 44	**1.233**	(166.54)	[1.230,1.236]	(0.000)
Age 45 to 64	**1**	(.)	[1,1]	(.)
**Race / Ethnicity**				
(nonrespondent)	**1.079**	(51.53)	[1.076,1.082]	(0.000)
Asian American, Native Hawaiian or Other Pacific Islander	**0.974**	(-15.08)	[0.971,0.978]	(0.000)
Black or African American	**1.387**	(103.72)	[1.379,1.396]	(0.000)
Latino	**1.108**	(66.80)	[1.105,1.112]	(0.000)
Other	**1.011**	(5.39)	[1.007,1.015]	(0.000)
White	**1**	(.)	[1,1]	(.)
**Language Spoken**				
Asian or Pacific Islander languages	**0.868**	(-56.44)	[0.864,0.872]	(0.000)
English	**1**	(.)	[1,1]	(.)
Other	**1.192**	(66.14)	[1.186,1.198]	(0.000)
Spanish	**0.946**	(-28.72)	[0.943,0.950]	(0.000)
**Health Plan Metal Tier**				
Minimum Coverage	**1.433**	(97.78)	[1.423,1.443]	(0.000)
Bronze	**1.113**	(88.99)	[1.111,1.116]	(0.000)
Silver	**1**	(.)	[1,1]	(.)
Gold / Platinum	**0.973**	(-16.58)	[0.970,0.977]	(0.000)
**Additional Factors**				
Broker Assistance	**0.859**	(-144.07)	[0.858,0.861]	(0.000)
Special enrollment period	**1.210**	(175.92)	[1.207,1.213]	(0.000)
Monthly premium $1 or less	**0.593**	(-290.31)	[0.591,0.596]	(0.000)
Household w/ Medicaid interaction	**0.735**	(-292.19)	[0.734,0.737]	(0.000)
Self-employment income	**0.530**	(-457.79)	[0.528,0.531]	(0.000)
**Gender**				
Female	**1**	(.)	[1,1]	(.)
Male	**0.999**	(-1.52)	[0.997,1.000]	(0.128)
**Household Size**				
No household size information	**0.965**	(-12.79)	[0.960,0.970]	(0.000)
1	**1**	(.)	[1,1]	(.)
2	**1.055**	(33.07)	[1.052,1.059]	(0.000)
3	**1.082**	(42.26)	[1.078,1.086]	(0.000)
4 or more	**1.062**	(44.79)	[1.059,1.065]	(0.000)
**Rating Region**				
Northern California	**1.013**	(9.11)	[1.010,1.016]	(0.000)
Bay Area	**1.038**	(25.45)	[1.035,1.040]	(0.000)
Los Angeles	**1**	(.)	[1,1]	(.)
Southern California	**1.055**	(40.97)	[1.052,1.057]	(0.000)
**N**	**6,521,763**			

© 2022 Wolf E et al. *JAMA Network Open.*

eTable 2. Relative Risk of Coverage Terminations (alternate models) – Panel E				
Model 5: Competing Risks Regression (on 10% sample)	**Competing Risks Regression** (Age>64 as Competing Risk)			
	**Subdistribution Hazard Ratio**	se	95% Confidence Interval	p-value
**Income Group** (as percent of Federal Poverty Level)				
150% FPL or less	**1.230**	(48.68)	[1.220,1.241]	(0.000)
150% FPL to 200% FPL	**1.033**	(8.58)	[1.025,1.040]	(0.000)
200% FPL to 400% FPL	**1**	(.)	[1,1]	(.)
400% FPL or greater or unsubsidized	**1.057**	(9.14)	[1.044,1.069]	(0.000)
**Age Group**				
Age 0 to 17	**1.551**	(72.95)	[1.533,1.569]	(0.000)
Age 18 to 29	**1.676**	(134.80)	[1.663,1.688]	(0.000)
Age 30 to 44	**1.615**	(127.70)	[1.603,1.627]	(0.000)
Age 45 to 64	**1**	(.)	[1,1]	(.)
**Race / Ethnicity**				
(nonrespondent)	**1.075**	(16.27)	[1.066,1.085]	(0.000)
Asian American, Native Hawaiian or Other Pacific Islander	**0.995**	(-0.93)	[0.986,1.005]	(0.350)
Black or African American	**1.401**	(34.93)	[1.375,1.428]	(0.000)
Latino	**1.133**	(27.88)	[1.123,1.143]	(0.000)
Other	**1.037**	(6.15)	[1.025,1.049]	(0.000)
White	**1**	(.)	[1,1]	(.)
**Language Spoken**				
Asian or Pacific Islander languages	**0.896**	(-15.29)	[0.884,0.909]	(0.000)
English	**1**	(.)	[1,1]	(.)
Other	**1.198**	(23.69)	[1.180,1.216]	(0.000)
Spanish	**0.970**	(-5.43)	[0.960,0.981]	(0.000)
**Health Plan Metal Tier**				
Minimum Coverage	**1.435**	(33.10)	[1.404,1.466]	(0.000)
Bronze	**1.130**	(34.56)	[1.122,1.138]	(0.000)
Silver	**1**	(.)	[1,1]	(.)
Gold / Platinum	**0.987**	(-2.60)	[0.978,0.997]	(0.009)
**Additional Factors**				
Broker Assistance	**0.867**	(-46.28)	[0.862,0.872]	(0.000)
Special enrollment period	**1.181**	(50.34)	[1.173,1.188]	(0.000)
Monthly premium $1 or less	**0.608**	(-96.96)	[0.602,0.614]	(0.000)
Household w/ Medicaid interaction	**0.780**	(-82.00)	[0.775,0.784]	(0.000)
Self-employment income	**0.587**	(-139.59)	[0.583,0.592]	(0.000)
**Gender**				
Female	**1**	(.)	[1,1]	(.)
Male	**0.997**	(-1.17)	[0.991,1.002]	(0.241)
**Household Size**				
No household size information	**0.994**	(-0.71)	[0.977,1.011]	(0.479)
1	**1**	(.)	[1,1]	(.)
2	**1.000**	(0.10)	[0.991,1.010]	(0.923)
3	**1.138**	(24.61)	[1.126,1.150]	(0.000)
4 or more	**1.082**	(20.47)	[1.074,1.091]	(0.000)
**Rating Region**				
Northern California	**0.988**	(-2.78)	[0.980,0.997]	(0.005)
Bay Area	**1.026**	(6.02)	[1.017,1.035]	(0.000)
Los Angeles	**1**	(.)	[1,1]	(.)
Southern California	**1.050**	(12.90)	[1.042,1.058]	(0.000)
**N**	**651,225**			

© 2022 Wolf E et al. *JAMA Network Open.*

eAppendix 2 - Details on the California Health Coverage Survey

Covered California Consumer Groups

The California Health Coverage Survey is an annual, web-based survey of Covered California consumers. The survey uses a probability-based sample and a mail-to-web design and is administered in English and Spanish. The sampling frames for each consumer group are directly from Covered California's administrative records and consist of heads of households aged 18-64 years old.

Survey respondents come from a probability-based sample of various Covered California consumer groups and the survey is designed to be representative of the following four consumer populations:

- Renewing members: consumers who were enrolled in a plan through Covered California in the year preceding the survey, and re-enrolled in Marketplace coverage during Open Enrollment in the year of the survey.
- Terminating members: consumers who were enrolled in a plan through Covered California in the year preceding the survey and did not re-enroll in Marketplace coverage during Open Enrollment in the year of the survey.
- New members: consumers who were not enrolled in a plan through Covered California in the year preceding the survey and enrolled in Marketplace coverage during Open Enrollment in the year of the survey.
- Funnel/Cancelled consumers: consumers who were not enrolled in a plan through Covered California in the year preceding the survey, but for the plan year of the survey had an application submission and were determined eligible for Covered California. Included in this group are consumers who made a plan selection during Open Enrollment but did not effectuate their Marketplace coverage.

For this study, we focus on two groups in particular: (1) those enrolled during Open Enrollment of the survey year ("new members"); and (2) those enrolled in a given year who ended their coverage in the year and did not re-enroll during Open Enrollment ("terminating members"). Due to concerns about respondent recall, the survey only sampled consumers who terminated after June 30th of the prior year; this sample is used to represent the full set of terminating members. Our comparisons of people who terminate before June 30th and after June 30th in Covered California's administrative data make us view this as a reasonable assumption.

The survey fielded in 2019 included a large oversample of terminating members, which provides a unique opportunity to analyze the subset of terminating members who went uninsured in that year.

Survey Response Rates

Our reporting follows the AAPOR guidelines for survey studies. All response rates shown are calculated using AAPOR RR1. Prior to sample selection, the sampling frame is limited to units that meet the eligibility requirements for the survey (head of household, 18-64 years old, and valid California mailing address), therefore all units in the survey sample are assumed to be eligible reporting units. Additionally, partial interviews are not counted as respondents and are instead counted as nonrespondents. Therefore, response rates are calculated as the number of completed surveys divided by the total number sampled. eTable 3 shows the response rate by survey cohort and for the overall survey, by year of survey fielding.

The fielding of the 2018 survey included a study to assess the impact of different methods of outreach and incentive structure on response rates, relying on repeated contact to increase response and representativeness of the survey.

Verification

Analysis of survey data relies on verified completes wherein a survey complete is considered verified if their self-reported enrollment status matches that of the administrative database. Verification rates for terminating members range from 81% to 84%, and 70% to 75% for new members.

© 2022 Wolf E et al. *JAMA Network Open.*

Exclusion of 2020 Survey Results

Survey data following the 2020 Open Enrollment period is excluded from this analysis due to unique circumstances related to the onset of the Covid-19 pandemic that raise concerns about data validity. Fielding of the 2020 survey coincided with the onset of the Covid-19 pandemic, and Covered California's establishment of a Special Enrollment Period to allow anyone impacted by the pandemic to enroll in coverage, unique circumstances that raise methodological concerns about validity of comparing 2020 survey data to the other years in this study.

Survey Question Wording

Current Coverage

Do you have health coverage right now?

1. Yes
2. No
3. Not sure

What is your main source of health coverage right now?

1. A plan through a current or former employer or union – of yours or a family member's (Includes COBRA)
2. A plan I bought on my own – or a family member bought on their own
   (Includes plans purchased through Covered California, an insurance company, an insurance agent, or a health insurance website)
3. Medi-Cal or Medicaid
   (Program that offers free or low-cost health coverage for children and adults with limited income and resources)
4. Medicare
   (Health insurance program for people 65 years and older or persons with certain disabilities)
5. TRICARE or other military health care
   (Includes VA health care)
6. A plan from another source
7. I do not have any health coverage right now.
8. Not sure

Previous source of coverage

For how many months in 2018 (2017) did you have health coverage? Your best guess is fine.

1. I never had it in 2018 (2017)
2. Less than 1 month
3. 1 month
4. 2 months
5. 3 months
6. 4 months
7. 5 months
8. 6 months
9. 7 months
10. 8 months
11. 9 months
12. 10 months
13. 11 months
14. All 12 months

© 2022 Wolf E et al. *JAMA Network Open.*

What was your main source of health coverage in 2018 (2017) [when you had it]?

1. A plan through a current or former employer or union – of yours or a family member's (Includes COBRA)
2. A plan I bought on my own – or a family member bought on their own
   (Includes plans purchased through Covered California, an insurance company, an insurance agent, or a health insurance website)
3. Medi-Cal or Medicaid
   (Program that offers free or low-cost health coverage for children and adults with limited income and resources)
4. Medicare
   (Health insurance program for people 65 years and older or persons with certain disabilities)
5. TRICARE or other military health care
   (Includes VA health care)
6. A plan from another source
7. Not sure

Additional Questions

How many times do you expect to go to a doctor's office or clinic to get health care for yourself this year? Think about the entire year (January 1 to December 31, 2019).

1. None
2. 1
3. 2
4. 3
5. 4
6. 5 to 9
7. 10 or more

Would you say the health plan you had through Covered California in 2018 was an excellent value, good value, only a fair value, or a poor value for what you paid for it?

1. Excellent
2. Good
3. Fair
4. Poor

What is the highest grade or level of school you have completed?

1. Did not complete high school
2. Graduated high school
3. Attended some college, but no degree
4. Graduated two-year college with Associate's degree
5. Graduated four-year college with Bachelor's degree
6. Obtained Master's, PhD, or other professional degree

© 2022 Wolf E et al. *JAMA Network Open.*

eTable 3. California Health Coverage Survey Response Rates by Consumer Cohort.

Cohort	Sample Size	Completed Surveys	Response Rate
**2018 Survey**			
New members	22,795	1,780	7.8%
Renewing members	45,229	3,473	7.7%
Terminating members	20,718	1,451	7.0%
Funnel and Cancelled members	35,700	2,385	6.7%
2018 Total	*124,442*	*9,089*	*7.3%*
**2019 Survey**			
New members	18,717	1,224	6.5%
Renewing members	17,737	1,675	9.4%
Terminating members	62,081	5,517	8.9%
Funnel and Cancelled members	46,586	3,396	7.3%
2019 Total	*145,121*	*11,812*	*8.1%*
**2021 Survey**			
New members	18,717	1,224	6.5%
Renewing members	17,737	1,675	9.4%
Terminating members	62,081	5,517	8.9%
Funnel and Cancelled members	46,586	3,396	7.3%
2021 Total	*145,121*	*11,812*	*8.1%*

eTable 4. Sample Distribution on Selected Database Demographic Variables Among 2017 Terminating Members.

Database Variable	Unweighted Sample*	Weighted Verified Sample	Population in Database
**Gender**			
Female	60.5%	53.7%	52.2%
Male	39.5%	46.3%	47.8%
**Race/Ethnicity**			
Asian/Pacific Islander	12.6%	12.8%	14.0%
Black or African American	4.0%	2.2%	2.5%
Latino	16.8%	22.9%	20.9%
Other	6.8%	8.8%	6.0%
White	40.8%	31.9%	32.5%
Unknown	19.0%	21.4%	24.2%

© 2022 Wolf E et al. *JAMA Network Open.*

Age			
18-29 years old	19.3%	25.6%	22.7%
30-44 years old	33.5%	34.6%	33.9%
45-64 years old	47.2%	39.8%	43.4%
**Federal Poverty Level**			
<138%	5.9%	6.0%	6.6%
138-200%	35.9%	40.4%	41.7%
200-400%	35.8%	34.3%	34.2%
>400%	8.0%	6.6%	6.2%
Missing	14.3%	12.7%	11.4%

*Note that some of the imbalances in the unweighted distribution reflect oversamples of key subgroups that are highly correlated with some of these demographic variables (e.g., Spanish-dominant consumers and consumers transitioning from Medicaid coverage).
**Note that FPL reported in this table is based on consumers' prior year FPL in the Database (the full sample was weighted, however, using current year FPL).

eTable 5. Sample Distribution on Select Database Demographic Variables Among 2018 Terminating Members.

Database Variable	Unweighted Sample*	Weighted Verified Sample	Population in Database
**Gender**			
Female	58.9%	53.2%	53.2%
Male	41.1%	46.8%	46.8%
**Race/Ethnicity**			
Asian/Pacific Islander	15.9%	14.6%	14.6%
Black or African American	3.3%	2.9%	2.9%
Latino	17.3%	22.5%	22.5%
Other	25.1%	29.0%	29.0%
White	38.4%	31.1%	31.1%
**Age**			
18-29 years old	19.5%	22.7%	22.7%
30-44 years old	33.1%	35.4%	35.4%
45-64 years old	47.4%	41.9%	41.9%
**Federal Poverty Level**			
<138%	4.5%	3.7%	3.7%
138-200%	37.1%	43.5%	43.3%
200-400%	39.0%	37.1%	37.2%
>400%	9.1%	6.3%	6.3%
Missing	10.3%	9.5%	9.5%

*Note that some of the imbalances in the unweighted distribution reflect oversamples of key subgroups that are highly correlated with some of these demographic variables (e.g., Spanish-dominant consumers and consumers transitioning from Medicaid coverage).
**Note that FPL reported in this table is based on consumers' prior year FPL in the Database (the full sample was weighted, however, using current year FPL).

eTable 6. Sample Distribution on Select Database Demographic Variables among 2020 Terminating Members.

Database Variable	Unweighted Sample*	Weighted Verified Sample	Population in Database
**Gender**			
Female	59.0%	53.1%	53.1%
Male	41.3%	46.9%	46.9%
**Race/Ethnicity**			
Asian/Pacific Islander	14.4%	15.0%	15.0%
Black or African American	10.3%	3.5%	3.5%
Latino	19.5%	23.2%	23.2%
Other	19.9%	27.5%	27.5%
White	36.0%	30.9%	30.9%
**Age**			
18-29 years old	22.9%	23.5%	23.5%
30-39 years old	24.5%	27.3%	27.3%
40-54 years old	24.3%	27.2%	27.2%
55-64 years old	28.4%	22.0%	22.0%
**Federal Poverty Level****			
<138%	3.6%	4.8%	4.8%
138-200%	37.2%	37.6%	40.2%
200-400%	43.7%	43.2%	40.6%
>400%	8.9%	7.8%	7.8%
Missing	6.7%	6.7%	6.7%

*Note that some of the imbalances in the unweighted distribution reflect oversamples of key subgroups that are highly correlated with some of these demographic variables (e.g., Spanish-dominant consumers and non-Hispanic Black consumers).
**Note that FPL reported in this table is based on consumers' prior year FPL in the Database.

eTable 7. Sociodemographic Characteristics of 2017 Terminating Members, by Current Source of Coverage.

	**All Terminating Members n=1,283**	**Uninsured Terminating Members n=130**	**Terminating Members with ESI n=598**	**Terminating Members with Medicaid n=271**	**Uninsured - ESI diff., *p***	**Uninsured-Medicaid diff., *p***	**ESI-Medicaid diff., *p***
**Age**							
Ages 18-29	26% (22%-29%)	17% (10%-25%)	28% (23%-34%)	25% (18%-34%)	0.05	0.24	0.48
Ages 30-44	35% (31%-39%)	42% (30%-55%)	39% (34%-45%)	26% (19%-35%)	0.67	0.04	0.01
Ages 45-64	40% (36%-44%)	41% (29%-53%)	33% (28%-38%)	49% (40%-58%)	0.24	0.3	0.003
**Race/Ethnicity**							
Asian/Pacific Islander	13% (10%-16%)	5% (3%-10%)	15% (12%-19%)	14% (9%-20%)	<0.001	0.01	0.77
Black	2% (1%-4%)	2% (1%-4%)	3% (2%-6%)	1% (0%-3%)	0.27	0.5	0.06
Latino	23% (19%-27%)	39% (28%-52%)	18% (14%-24%)	27% (19%-36%)	0.003	0.11	0.12
Other	9% (7%-12%)	5% (2%-16%)	10% (7%-14%)	6% (2%-13%)	0.19	0.91	0.17
White	32% (28%-35%)	25% (16%-36%)	35% (30%-40%)	27% (21%-34%)	0.09	0.78	0.06
Unknown	21% (18%-25%)	24% (14%-37%)	18% (14%-23%)	26% (18%-35%)	0.4	0.77	0.12
**FPL**							
Under 200% FPL	46% (42%-50%)	49% (37%-61%)	40% (35%-46%)	62% (53%-71%)	0.21	0.09	<0.001
200% - 400% FPL	35% (31%-39%)	37% (26%-49%)	36% (31%-41%)	27% (20%-37%)	0.84	0.2	0.11
Over 400% FPL/Unsubsidized	19% (16%-22%)	14% (8%-24%)	24% (20%-29%)	10% (6%-17%)	0.03	0.44	<0.001
**Education**							
No college degree	41% (37%-45%)	67% (55%-77%)	29% (24%-35%)	54% (45%-63%)	<0.001	0.08	<0.001
College degree	59% (55%-63%)	33% (23%-45%)	71% (65%-76%)	46% (37%-55%)	<0.001	0.08	<0.001
**Region**							
Los Angeles	27% (23%-31%)	31% (31%-43%)	37% (26%-37%)	16% (11%-22%)	0.98	0.02	<0.001
Southern California	31% (27%-35%)	26% (17%-37%)	27% (22%-33%)	42% (33%-51%)	0.77	0.03	0.008
Bay Area	23% (20%-26%)	15% (9%-25%)	25% (21%-30%)	23% (16%-31%)	0.03	0.18	0.58
Northern California	19% (16%-23%)	28% (18%-42%)	17% (13%-21%)	20% (14%-28%)	0.08	0.27	0.36
**Metal Tier**							
Bronze/Catastrophic	38% (34%-42%)	40% (29%-53%)	43% (38%-49%)	29% (21%-37%)	0.65	0.12	0.004
Silver 70/73	22% (19%-26%)	19% (12%-29%)	21% (17%-25%)	22% (15%-31%)	0.77	0.65	0.78
Silver 87/94	32% (28%-36%)	37% (25%-50%)	26% (22%-32%)	47% (38%-56%)	0.13	0.22	<0.001
Gold/Platinum	8% (6%-10%)	4% (1%-12%)	10% (7%-14%)	3% (1%-8%)	0.05	0.72	0.004

Note: The “Other” Race/Ethnicity category includes individuals who reported their race as American Indian/Alaskan Native, Multiple Races, or Other. Asian/Pacific Islander category also includes individuals who report their race as Native Hawaiian

eTable 8. Sociodemographic Characteristics of 2018 Terminating Members, by Current Source of Coverage.

	All Terminating Members n=4,517	Uninsured Terminating Members n=875	Terminating Members with ESI n=1,674	Terminating Members with Medcaid n=874	Uninsured -ESI diff., *p*	Uninsured -Medicaid diff., *p*	ESI-Medicaid diff., *p*
**Age**							
Ages 18-29	23% (20%-26%)	23% (17%-30%)	25% (21%-29%)	24% (19%-31%)	0.62	0.83	0.8
Ages 30-44	35% (33%-38%)	30% (23%-36%)	42% (38%-47%)	30% (24%-36%)	0.001	0.95	0.001
Ages 45-64	42% (39%-45%)	48% (20%-55%)	33% (29%-37%)	46% (40%-53%)	<0.001	0.81	<0.001
**Race/Ethnicity**							
Asian/Pacific Islander	15% (13%-17%)	13% (9%-18%)	19% (16-23%)	11% (8%-15%)	0.02	0.49	<0.001
Black	3% (2%-4%)	3% (2%-5%)	2% (2%-4%)	4% (3%-7%)	0.53	0.4	0.12
Latino	23% (20%-25%)	40% (33%-47%)	18% (15%-22%)	25% (19%-31%)	<0.001	0.002	0.07
Other	9% (7%-11%)	6% (3%-10%)	7% (5%-10%)	15% (10%-21%)	0.57	0.005	0.009
White	31% (28%-34%)	24% (19-30%)	32% (28%-36%)	33% (27%-40%)	0.03	0.04	0.72
Unknown	19% (17%-22%	15% (10%-20%)	21% (18%-25%)	12% (8%-18%)	0.04	0.5	0.004
**FPL**							
Under 200% FPL	47% (44%-50%)	53% (46%-60%)	37% (33%-42%)	73% (66%-78%)	<0.001	<0.001	<0.001
200% - 400% FPL	37% (34%-40%)	36% (29%-43%)	43% (39%-47%)	23% (18%-29%)	0.07	0.007	<0.001
Over 400% FPL/Unsubsidized	16% (14%-18%)	11% (7%-17%)	20% (17%-23%)	4% (2%-7%)	0.003	0.006	<0.001
**Education**							
No college degree	44% (41%-47%)	71% (64%-77%)	31% (27%-35%)	53% (46%-60%)	<0.001	<0.001	<0.001
College degree	56% (53%-59%)	29% (23%-36%)	69% (65%-73%)	47% (40%-54%)	<0.001	<0.001	<0.001
**Region**							
Los Angeles	27% (24%-30%)	30% (24%-37%)	29% (25%-34%)	21% (16%-27%)	0.82	0.04	0.02
Southern California	34% (31%-37%)	37% (31%-45%)	26% (22%-30%)	40% (34%-47%)	0.005	0.55	<0.001
Bay Area	21% (19%-24%)	11% (8%-16%)	26% (23%-30%)	22% (17%-28%)	<0.001	0.004	0.16
Northern California	18% (16%-20%)	21% (16%-27%)	18% (15%-22%)	17% (12%-23%)	0.46	0.32	0.66
**Metal Tier**							
Bronze/Catastrophic	35% (33%-38%)	35% (29%-42%)	43% (39%-48%)	23% (18%-29%)	0.04	0.005	<0.001
Silver 70/73	15% (13%-17%)	15% (10%-21%)	14% (11%-17%)	7% (4%-12%)	0.69	0.02	0.007
Silver 87/94	34% (32%-37%)	39% (32%-46%)	25% (21%-29%)	57% (50%-63%)	0.001	<0.001	<0.001
Gold/Platinum	16% (14%-18%)	11% (7%-17%)	18% (15%-22%)	13% (9%-19%)	0.02	0.49	0.12

Note: The “Other” Race/Ethnicity category includes individuals who reported their race as American Indian/Alaskan Native, Multiple Races, or Other. Asian/Pacific Islander category also includes individuals who report their race as Native Hawaiian.

© 2022 Wolf E et al. *JAMA Network Open.*

eTable 9. Sociodemographic Characteristics of 2020 Terminating Members, by Current Source of Coverage.

	All Terminating Members n=674	Uninsured Terminating Members n=81	Terminating Members with ESI n=302	Terminating Members with Medi-Cal n=193	Uninsured - ESI diff., *p*	Uninsured-Medicaid diff., *p*	ESI-Medicaid diff., *p*
**Age**							
Ages 18-29	24% (20%-27%)	26% (17%-39%)	26% (21%-31%)	25% (19%-32%)	0.94	0.88	0.91
Ages 30-44	36% (32%-41%)	29% (19%-41%)	46% (39%-52%)	29% (23%-37%)	0.009	0.95	0.001
Ages 45-64	40% (36%-44%)	45% (34%-57%)	29% (23%-35%)	46% (38%-53%)	0.02	0.93	0.001
**Race/Ethnicity**							
Asian/Pacific Islander	15% (12%-18%)	7% (3%-15%)	17% (13%-22%)	16% (11%-23%)	0.01	0.04	0.82
Black	3% (3%-5%)	4% (2%-8%)	4% (2%-6%)	3% (2%-5%)	0.86	0.44	0.36
Latino	23% (19%-27%)	39% (28%-52%)	20% (15%-25%)	26% (20%-33%)	0.004	0.07	0.14
Other	8% (6%-11%)	1% (0%-8%)	8% (5%-12%)	12% (8%-19%)	0.005	<0.001	0.17
White	31% (27%-35%)	30% (20%-41%)	32% (27%-38%)	26% (20%-32%)	0.75	0.51	0.15
Unknown	20% (16%-24%)	19% (11%-31%)	20% (15%-27%)	17% (12%-24%)	0.78	0.81	0.45
**FPL**							
Under 200% FPL	42% (38%-47%)	48% (36%-60%)	32% (27%-39%)	60% (53%-67%)	0.03	0.09	<0.001
200% - 400% FPL	43% (39%-47%)	48% (36%-60%)	46% (40%-52%)	34% (27%-41%)	0.81	0.06	0.01
Over 400% FPL/Unsubsidized	14% (12%-18%)	5% (2%-12%)	22% (17%-27%)	6% (3%-11%)	<0.001	0.65	<0.001
**Education**							
No college degree	46% (42%-50%)	74% (63%-83%)	35% (29%-41%)	53% (46%-61%)	<0.001	0.001	<0.001
College degree	54% (50%-58%)	26% (13%-36%)	65% (59%-71%)	47% (39%-54%)	<0.001	0.001	<0.001
**Region**							
Los Angeles	29% (25%-32%)	30% (21%-42%)	31% (25%-37%)	24% (19%-32%)	0.97	0.36	0.17
Southern California	31% (27%-35%)	40% (28%-52%)	27% (21%-33%)	34% (28%-42%)	0.06	0.47	0.09
Bay Area	22% (19%-25%)	5% (2%-12%)	28% (23%-34%)	18% (13%-25%)	<0.001	0.001	0.01
Northern California	19% (15%-22%)	25% (15%-37%)	15% (11%-20%)	23% (17%-31%)	0.11	0.81	0.05
**Metal Tier**							
Bronze/Catastrophic	37% (33%-41%)	44% (32%-56%)	44% (38%-50%)	28% (22%-35%)	0.95	0.03	0.001
Silver 70/73	19% (16%-23%)	15% (8%-26%)	21% (16%-26%)	13% (9%-19%)	0.26	0.7	0.04
Silver 87/94	31% (27%-35%)	25% (16%-37%)	24% (19%-30%)	46% (38%-54%)	0.85	0.002	<0.001
Gold/Platinum	13% (11%-16%)	16% (9%-27%)	11% (8%-16%)	13% (9%-19%)	0.32	0.52	0.62

Note: The “Other” Race/Ethnicity category includes individuals who reported their race as American Indian/Alaskan Native, Multiple Races, or Other. Asian/Pacific Islander category also includes individuals who report their race as Native Hawaiian.

© 2022 Wolf E et al. *JAMA Network Open.*

eAppendix 3. Modeling Terminations Who Go Uninsured

While uninsured terminating consumers largely cite costs and affordability as their main reasons for not re-enrolling (eFigure 2), changes in net premium do not provide any explanatory evidence in our model of those who terminate to go uninsured. We ran different iterations of the model, looking at changes in gross premium, before applying premium subsidies, (eTable 11) and changes in net premium only among those end-of-year consumers who had updated eligibility information processed (eTable 12). In neither model was there a significant effect on renewal candidates' decision to churn or renew coverage. Prices and affordability are concerns for all consumers, but this modeling indicates that their relative importance among uninsured terminating consumers diminishes when accounting for other affordability factors of plan value and expected utilization.

eFigure 2. Self-Reported Reasons for Terminating Coverage, Among Uninsured Renewal Candidates.

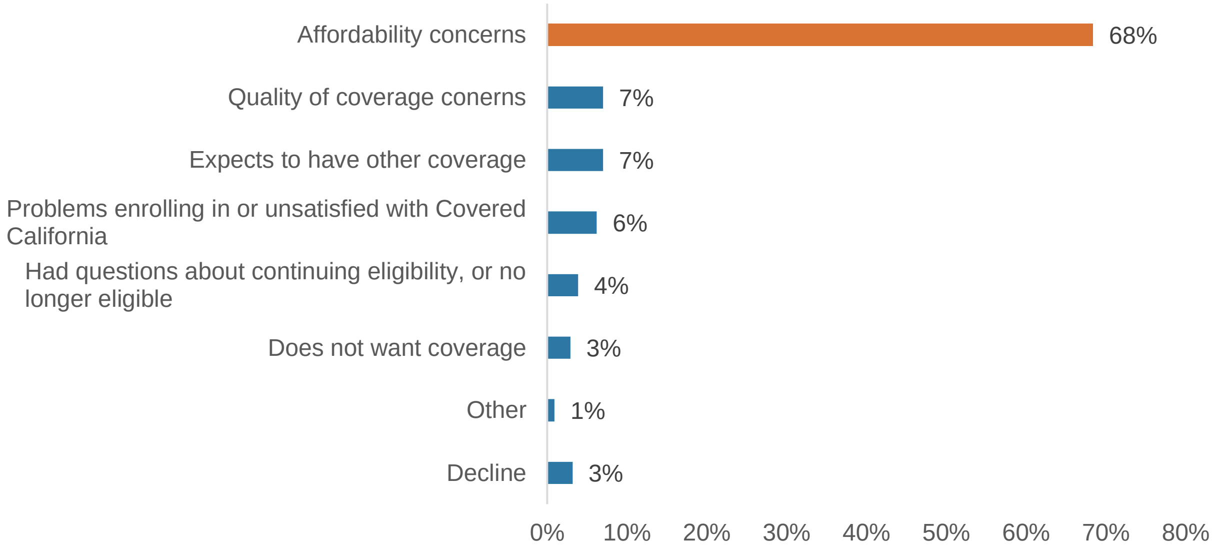

Source: California Health Coverage Survey. N=645; 95% Confidence Intervals for key estimates: Affordability: 63%-73%; Quality: 5%-10%; Other coverage: 4%-12%; Covered California: 6%-12%.

© 2022 Wolf E et al. *JAMA Network Open.*

eTable 10. Observations of Independent Variables Among Subsidized Renewal Candidates

**Subsidized Renewal Candidates**	**N=1,621**
**Penalty awareness**	
Unaware	864
Aware*	757
**Expected number of doctor visits**	
None	275
One or more*	1,346
**Race/Ethnicity**	
Asian/Pacific Islander	357
Black or African American	166
Latino	546
Other	44
White *	492
Unknown	16
**2018 Insurance status**	
Insured all year*	1,347
Uninsured part of year	274
**Health plan value rating**	
Excellent/Good/Fair*	1,387
Poor	234
**FPL**	
138%-200% FPL*	927
200%-300% FPL	480
300%-400% FPL	214
**Metal Tier**	
Bronze	619
Silver	830
Gold/Platinum*	172
**Education**	
No college degree	971
College degree*	651
**Age Range**	
Ages 18-29	230
Ages 30-44	419
Ages 45-64*	972
**Region**	
Los Angeles	452
Southern California	580
Bay Area	288
Northern California*	301

Source**:** California Health Coverage Survey and Covered California administrative data.
Notes: * indicates base category in logistic regression model. The "Other" Race/Ethnicity category includes individuals who reported their race as American Indian/Alaskan Native, Multiple Races, or Other. Asian/Pacific Islander category also includes individuals who report their race as Native Hawaiian.

© 2022 Wolf E et al. *JAMA Network Open.*

eTable 11. Logistical Regression Modeling Output: Decision to Renew or Terminate and Go Uninsured Among Subsidized Renewal Candidates

	Coef.	se	t	P>t	95% Confidence Interval	
**Aware of penalty repeal**	1.334	0.222	6.000	0.000	0.898	1.771
**Percent change in net premium**	-0.965	1.800	-0.540	0.592	-4.495	2.566
**Expected number of doctor visits**						
None	1.974	0.275	7.190	0.000	1.436	2.513
**Race/Ethnicity**						
Asian/Pacific Islander	0.226	0.299	0.760	0.450	-0.360	0.812
Black	0.615	0.455	1.350	0.177	-0.278	1.508
Latino	1.186	0.258	4.590	0.000	0.680	1.693
Other	0.492	0.494	1.000	0.320	-0.477	1.461
Unknown	1.420	0.655	2.170	0.030	0.135	2.704
**Uninsured at any point in 2018**	0.989	0.276	3.580	0.000	0.447	1.531
**Value rating of health plan**						
Poor	1.121	0.294	3.810	0.000	0.544	1.698
**Metal Tier**						
Bronze	-0.354	0.357	-0.990	0.322	-1.055	0.347
Silver	-0.021	0.332	-0.060	0.950	-0.671	0.630
**FPL**						
200-300% FPL	0.280	0.262	1.070	0.286	-0.235	0.794
300-400% FPL	0.974	0.306	3.180	0.001	0.373	1.575
**Education**						
No college degree	0.556	0.241	2.310	0.021	0.084	1.028
**Age**						
18-29	0.202	0.312	0.650	0.517	-0.409	0.813
30-44	-0.087	0.294	-0.300	0.766	-0.664	0.489
**Region**						
Los Angeles	0.711	0.340	2.090	0.037	0.043	1.379
Southern California	0.699	0.295	2.370	0.018	0.120	1.278
Bay Area	0.266	0.382	0.700	0.487	-0.484	1.015
**Constant**	-7.235	0.523	-13.830	0.000	-8.261	-6.209

Source**:** Authors' analysis of California Health Coverage Survey data supplemented with Covered California administrative data. N = 1,621; DV = terminate coverage and go uninsured. The "Other" Race/Ethnicity category includes individuals who reported their race as American Indian/Alaskan Native, Multiple Races, or Other. Asian/Pacific Islander category also includes individuals who report their race as Native Hawaiian.

© 2022 Wolf E et al. *JAMA Network Open.*

eTable 12. Average Marginal Effects of Decision to Renew or Terminate and Go Uninsured (Alternative Specification Using Gross Premium Rate Changes Instead of Net-of-Subsidy Rate Changes)

	Effect size	se	p-value	95% Confidence Interval	
**Aware of penalty repeal**	0.017	0.004	0.000	0.010	0.024
**Percent change in gross premium**	0.016	0.016	0.327	-0.016	0.048
**Expected number of doctor visits**					
None	0.050	0.013	0.000	0.025	0.076
**Race/Ethnicity**					
Asian/Pacific Islander	0.002	0.002	0.456	-0.003	0.007
Black	0.006	0.006	0.252	-0.005	0.018
Latino	0.015	0.004	0.000	0.008	0.023
Other	0.005	0.005	0.369	-0.006	0.016
**Uninsured at any point in 2018**	0.015	0.006	0.009	0.004	0.027
**Value rating of health plan**					
Poor	0.018	0.007	0.013	0.004	0.032
**Metal Tier**					
Bronze	-0.005	0.005	0.264	-0.015	0.004
Silver	0.000	0.005	0.987	-0.009	0.009
**FPL**					
200-300% FPL	0.003	0.003	0.333	-0.003	0.009
300-400% FPL	0.016	0.006	0.011	0.004	0.028
**Education**					
No college degree	0.006	0.003	0.019	0.001	0.012
**Age**					
18-29	0.004	0.004	0.397	-0.005	0.012
30-44	-0.001	0.003	0.863	-0.007	0.006
**Region**					
Los Angeles	0.007	0.004	0.069	-0.001	0.014
Southern California	0.007	0.003	0.014	0.002	0.013
Bay Area	0.002	0.003	0.528	-0.005	0.009
**Expected utilization over value**					
**No visits**					
Excellent/Good/Fair	0.045	0.012	0.000	0.021	0.070
Poor	0.113	0.030	0.000	0.054	0.172

Source**:** Authors' analysis of California Health Coverage Survey supplemented with administrative data from Covered California. N = 1,621. The "Other" Race/Ethnicity category includes individuals who reported their race as American Indian/Alaskan Native, Multiple Races, or Other. Asian/Pacific Islander category also includes individuals who report their race as Native Hawaiian.

© 2022 Wolf E et al. *JAMA Network Open.*

eTable 13. Average Marginal Effects of Decision to Renew or Terminate and Go Uninsured (Alternative Specification Among Subsidized Renewal Candidates With Updated Eligibility During 2019 Open Enrollment)

	Effect size	se	p-value	95% Confidence Interval	
**Penalty awareness**	0.014	0.005	0.002	0.005	0.023
**Percent change in net premium**	-0.021	0.029	0.475	-0.078	0.036
**Expected number of doctor visits**					
None	0.041	0.015	0.005	0.012	0.069
**Race/Ethnicity**					
Asian/Pacific Islander	0.002	0.003	0.500	-0.004	0.009
Black	0.012	0.011	0.258	-0.009	0.033
Latino	0.020	0.006	0.001	0.008	0.032
Other	0.004	0.007	0.620	-0.011	0.018
**Uninsured at any point in 2018**	0.016	0.009	0.063	-0.001	0.034
**Value rating of health plan**					
Poor	0.012	0.008	0.173	-0.005	0.028
**Metal Tier**					
Bronze	0.006	0.004	0.169	-0.003	0.015
Silver	0.009	0.004	0.027	0.001	0.016
**FPL**					
200-300% FPL	0.004	0.005	0.434	-0.006	0.014
300-400% FPL	0.023	0.010	0.020	0.004	0.043
**Education**					
No college degree	0.007	0.004	0.093	-0.001	0.015
**Age**					
18-29	-0.001	0.006	0.905	-0.012	0.011
30-44	-0.002	0.005	0.639	-0.012	0.007
**Region**					
Los Angeles	0.001	0.006	0.897	-0.011	0.012
Southern California	0.005	0.005	0.341	-0.005	0.015
Bay Area	-0.003	0.005	0.491	-0.015	0.007
**Expected utilization over value**					
**No visits**					
Excellent/Good/Fair	0.037	0.014	0.009	0.010	0.065
Poor	0.074	0.026	0.004	0.024	0.124

Source: Authors' analysis of California Health Coverage Survey data supplemented with Covered California administrative data. N=815. The "Other" Race/Ethnicity category includes individuals who reported their race as American Indian/Alaskan Native, Multiple Races, or Other. Asian/Pacific Islander category also includes individuals who report their race as Native Hawaiian.

References

1. Austin PC, Lee DS, Fine JP. Introduction to the Analysis of Survival Data in the Presence of Competing Risks. *Circulation*. 2016;133:601-609.

2. Fine JP, Gray RJ. A Proportional Hazards Model for the Subdistribution of a Competing Risk. *J Am Stat Assoc*. 1999;94(446):496-509. doi:10.1080/01621459.1999.10474144

© 2022 Wolf E et al. *JAMA Network Open.*
