# Supplementary material for: Assessment of Churn in Coverage Among California’s Health Insurance Marketplace Enrollees
Source: JAMA Health Forum. 2022 Dec 2;3(12):e224484. doi: 10.1001/jamahealthforum.2022.4484 (PMC9719048; doi:10.1001/jamahealthforum.2022.4484)
Supplement: Supplement 2. — Data Sharing Statement [file jamahealthforum-e224484-s002.pdf]

Data Sharing Statement

Wolf. Assessment of Churn in Coverage Among California's Health Insurance Marketplace Enrollees. *JAMA Health Forum*. Published December 02, 2022.
doi:10.1001/jamahealthforum.2022.4484

Data
Data available: Yes
Data types: Deidentified participant data
How to access data: Deidentified data can be made available upon request. Requests for more information can be sent to Emory Wolf (emory.wolf@covered.ca.gov).
When available: With publication

Supporting Documents
Document types: None

Additional Information
Who can access the data: Data will be made available to anyone submitting a request.
Types of analyses: For any purposes.
Mechanisms of data availability: Data will be made available after approval of a proposal.
